# Fluorescence Tuning of Carbon Dots from Red to Blue via UV‐Induced Photochemical Etching

**DOI:** 10.1002/advs.75625

**Published:** 2026-05-08

**Authors:** Nanzhi Zheng, Yuchen Yang, Jingwei Xu, Jincheng Gan, Yinuo An, Xinyu Li, Guohua Chen

**Affiliations:** ^1^ College of Materials Science and Engineering Huaqiao University Xiamen P. R. China; ^2^ Graphene Powder & Composite Materials Research Center of Fujian Province The Xiamen Key Laboratory of Polymers & Electronic Materials Huaqiao University Xiamen P. R. China

**Keywords:** carbon dots, fluorescence tuning, information encryption, multicolor emission, photochemical etching

## Abstract

Carbon dots (CDs) have attracted extensive interest owing to their outstanding optical properties, biocompatibility, and low‐cost synthesis. Nevertheless, the preparation of multicolor CDs remains complex and most post‐synthetic strategies allow only limited wavelength modulation, the full‐spectrum tunability across the visible region still has yet to be achieved. Herein, we report a simple photochemical strategy that enables continuous fluorescence tuning of red‐emissive CDs (R‐CDs) via controlled UV‐induced photoetching in N,N‐dimethylformamide. UV irradiation generates reactive radical species, which progressively disrupt graphitic domains. Meanwhile, solvent–surface interactions synchronously regulate emissive surface states, and the two effects together enable precise size control and continuous emission modulation. Consequently, the emission peak of the R‐CDs exhibits a systematic blue shift from 657 nm to 573, 530, 490, and ultimately 443 nm, achieving full‐spectrum emission across the visible range. Notably, irradiation time serves as a single adjustable parameter, greatly simplifying the optimization of multiple synthetic conditions. Beyond their excellent performance in high–color‐rendering‐index white light‐emitting diodes, the photochromic CDs were further applied in multi‐level fluorescent information encryption, achieving five distinct levels of optical security. This work establishes a robust platform for progressive fluorescence tuning of CDs with promising potential in advanced photonic and information‐security applications.

## Graphical abstract

A UV‐driven photochemical process enables continuous color evolution of carbon dots from red to blue using a single irradiation parameter. Progressive photoetching restructures graphitic domains and surface states, allowing full visible‐spectrum emission control. The resulting multicolor carbon dots support high‐quality white light emission and multilevel optical encryption applications.

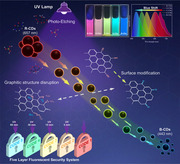

## 1 Introduction

Carbon dots (CDs), a novel class of fluorescent nanomaterials, have garnered significant interest owing to their excellent photoluminescent properties, high biocompatibility, and potential applications in bioimaging [1, 2, 3], optoelectronic devices [4, 5], information encryption [6, 7], and energy conversion [8, 9]. Compared with traditional semiconductor quantum dots, CDs not only exhibit quantum confinement effects but also possess abundant surface functional groups [10]. Their fluorescence originates from various mechanisms, including core states [11], surface states [12], molecular states [13], and cross‐linked enhanced emission [14], providing diverse pathways for fluorescence modulation. Depending on the synthesis route of the CDs, corresponding strategies for tuning their photoluminescence can be selected.

CDs are typically synthesized via two general strategies: top‐down and bottom‐up [15]. The top‐down approach involves breaking down bulk carbon materials (e.g., graphite [16], carbon black [17], or carbon nanotubes [18]) into nanoscale CDs through strong acid oxidation, electrochemical treatment, or laser ablation. Although it allows partial control over particle size and edge chemistry, this approach suffers from limited tunability due to the chemical inertness of precursors and the harsh reaction conditions required. In contrast, bottom‐up synthesis enable better control over particle size, heteroatom doping, and carbonization degree by rationally designing molecular precursors and adjusting reaction parameters [19]. However, CDs obtained from a single reaction system generally exhibit monochromatic emission. Achieving multicolor fluorescence typically requires altering reaction conditions—such as the precursor type [20], temperature [21], dopant [22], or catalyst [23] —across multiple syntheses, resulting in complex and poorly controllable processes. Although one‐pot syntheses can yield mixtures of CDs with varied emissions, the resulting colors are usually discrete and require tedious post‐synthetic separation [24, 25, 26]. Beyond synthetic routes, post‐treatment approaches—including environmental modulation (e.g., changes in pH or solvent) [27], aggregation control [28], and surface modification (e.g., passivation, oxidation, or grafting) [29, 30] —have also been explored to tune optical properties. Although such treatments can fine‐tune fluorescence characteristics, their regulation precision remains insufficient, making it difficult to achieve continuous wavelength shifts or controlled fluorescence modulation. Thus, realizing efficient, continuously tunable, and precisely controllable multicolor emission within a single CD system remains a key challenge.

Photo‐induced modulation has recently emerged as a promising alternative for fluorescence control. Several studies have demonstrated that photon energy can modify the surface fluorophores and oxygen defect states of CDs, leading to fluorescence transitions—for instance, from red to green—under ultraviolet (UV) irradiation, with applications in UV sensing [31]. Gao et al. prepared two kinds of photochromic CDs through a sodium doping strategy, which display color‐tunable fluorescence under ultraviolet irradiation [32]. Zhang et al. synthesized green‐emitting CDs exhibiting photochromic responsiveness in ammonia solution; upon irradiation, their fluorescence changes from green to orange [33]. Furthermore, some researchers have fabricated CDs‐polymer composite materials, which achieve fluorescence conversion under stimulation by reactive oxygen species/active free radicals generated upon light exposure [34, 35]. Although light‐induced modulation shows promise, these studies mostly focus on binary color transitions and do not explore strategies for achieving continuous and progressive multicolor tuning in a unified system. Inspired by top‐down exfoliation and fragmentation approaches for carbon materials, CDs—which typically possess a higher density of defects, heteroatoms, and surface functional groups—exhibit inferior coplanarity in their graphitic layers compared to graphite. This structural characteristic facilitates their further exfoliation and etching under relatively mild conditions. Previous studies have demonstrated that relatively large CDs can be exfoliated into monolayers under microwave‐assisted conditions, resulting in a red‐to‐near‐infrared fluorescence shift [36]. Similarly, UV irradiation can gradually reduce CD size and mass with increasing exposure time and intensity, indicating structural degradation, though without a notable spectral shift [37]. Moreover, alkali‐assisted photo‐irradiation has been shown to exfoliate multilayer CDs into monolayers, accompanied by a fluorescence change from red to green, yet such discrete changes fall short of true multicolor control [38]. These findings suggest that photoinduced etching could be a viable route to modulate CD fluorescence finely.

In this work, a controllable photochemical strategy is presented for continuously tuning CD fluorescence via UV‐induced photochemical etching (photoetching). In this study, large‐sized, red‐emissive nitrogen‐doped CDs (R‐CDs) were synthesized through a solvothermal method and dispersed in N, N‐dimethylformamide (DMF). Upon UV irradiation, the R‐CDs exhibited a continuous blue shift in emission, with the fluorescence peak shifting sequentially from 657 nm to 573, 530, 490, and ultimately 443 nm. The resulting orange (O‐CDs), yellow (Y‐CDs), green (G‐CDs), and blue (B‐CDs) exhibit a continuous multicolor emission transition within a single reaction system. Mechanistic investigations, including structural evolution analysis and inhibition experiments using trifluoroacetic acid (TFA) and ascorbic acid (AA), revealed that photoinduced radical species break the carbon framework and etch the graphitic domains. Simultaneously, reactions with the solvent induce surface state modifications. This synergistic process enables precise size control and continuous modulation of the emission wavelength through a straightforward, time‐dependent irradiation approach. Furthermore, the dynamically color‐switchable CDs were successfully employed in advanced fluorescence‐based encryption systems. By selectively controlling the UV exposure time, up to five levels of optical information encryption were achieved—far surpassing the capabilities of conventional dual‐mode systems based on fluorescence and room‐temperature phosphorescence. This work not only demonstrates a highly controllable and efficient strategy for fluorescence tuning in CDs but also opens new pathways for their application in multifunctional optoelectronic and information security technologies.

## 2 Results and Discussion

### 2.1 Morphologies and Structures of CDs

**FIGURE 1:**
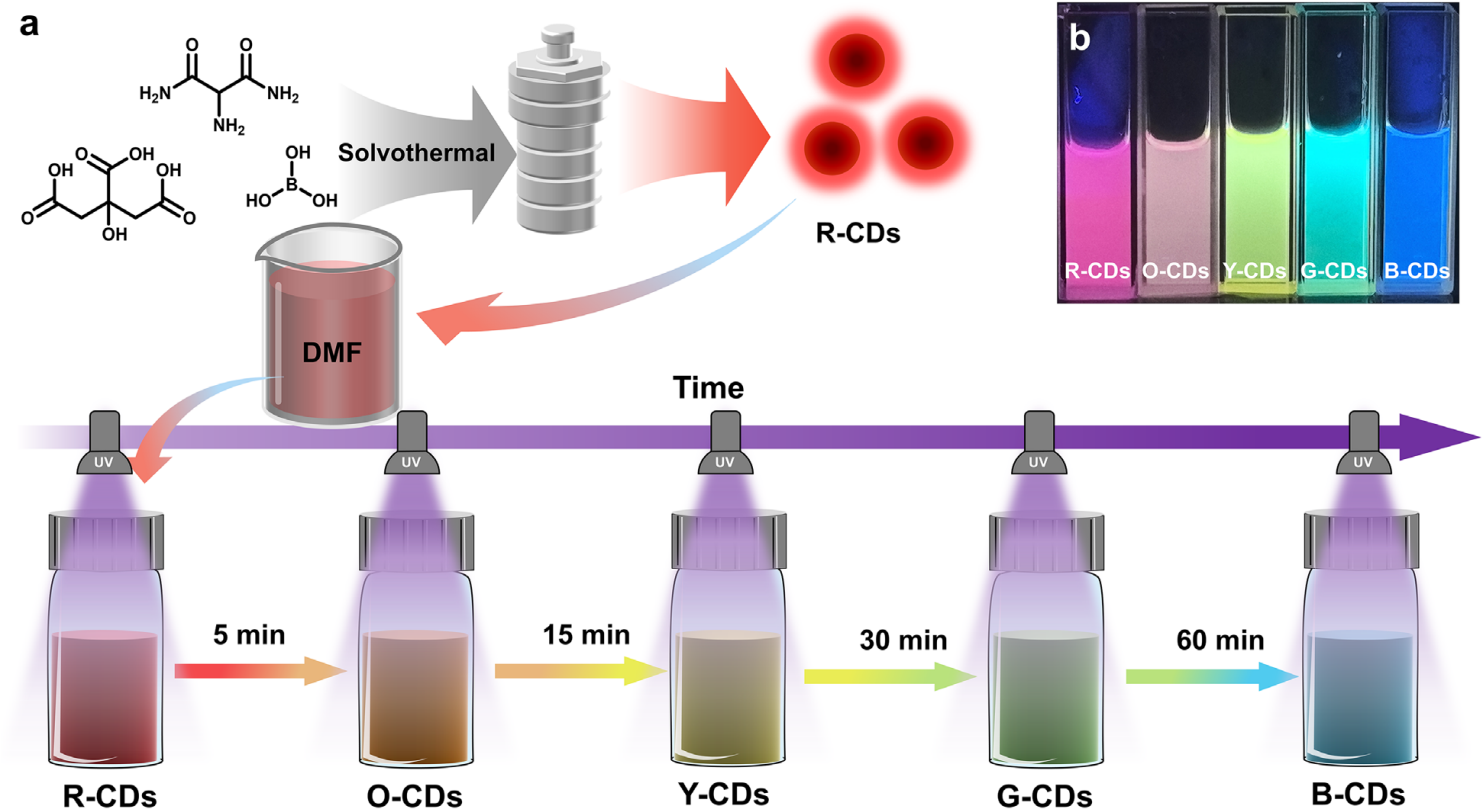
(a) Synthesis process of multicolor CDs. (b) Photographs of multicolor CDs under 365 nm UV light.

As illustrated in Figure 1a, R‐CDs with strong fluorescence were synthesized via a solvothermal method using citric acid, 2‐aminomalonamide, and boric acid as precursors. As shown in Figure S2, R‐CDs dissolved in various solvents exhibit varying degrees of blue shift after UV irradiation, while in DMF, the R‐CDs display the optimal solubility and the highest fluorescence intensity, and most importantly, the blue shift is the most pronounced in DMF, which allows the R‐CDs to be tuned over a wide range. The normalized fluorescence spectra of R‐CDs in DMF solution after different UV irradiation durations are displayed in Figure S4. The R‐CDs exhibit a continuous blue shift, and the fluorescence peak position stabilizes after 60 min of irradiation. The solution concentration was 0.03 mg/mL. Besides prolonging the irradiation time, a higher concentration primarily leads to inhomogeneous photoreaction and the formation of mixed CDs with multiple coexisting sizes. As shown in Figure S3, the CDs solution only emits mixed white fluorescence even with prolonged irradiation. Aliquots were collected at 5, 15, 30, and 60 min of UV exposure and purified via column chromatography, yielding multicolor CDs—O‐CDs, Y‐CDs, G‐CDs, and B‐CDs—emitting orange, yellow, green, and blue fluorescence, respectively.

**FIGURE 2:**
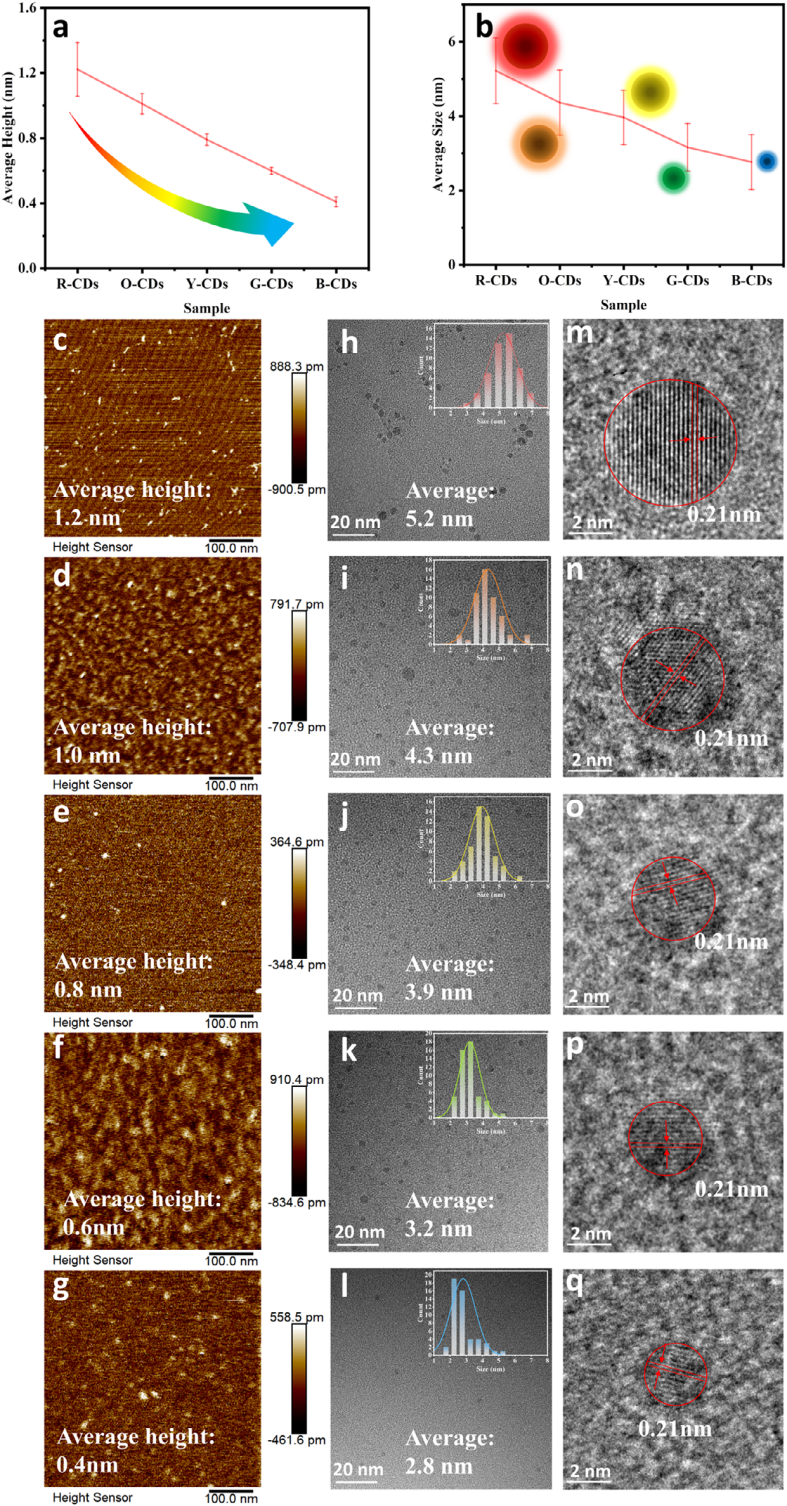
(a) Variation in the average height of multicolor CDs. (b) Variation in the average size of multicolor CDs. AFM image of R‐CDs (c), O‐CDs (d), Y‐CDs (e), G‐CDs (f), and B‐CDs (g). Transmission electron microscopy (TEM) images of R‐CDs (h), O‐CDs (i), Y‐CDs (j), G‐CDs (k), and B‐CDs (l) and their particle size distribution. High‐resolution TEM images of R‐CDs (m), O‐CDs (n), Y‐CDs (o), G‐CDs (p), and B‐CDs (q).

To elucidate the photoetching mechanism responsible for the fluorescence blue‐shift, we systematically characterized the morphological and structural changes of CDs at different irradiation stages (R‐CDs to B‐CDs). Figure 2a,b present the variations in the average height and lateral size of the multicolor CDs. With increasing UV irradiation time, the fluorescence emission of the CDs exhibits a continuous blue shift, accompanied by a significant decrease in both height and size. This trend is strongly correlated with the quantum size effect. Atomic force microscopy (AFM) images (Figure 2c–g) and Height Curves of the Selected Regions (Figures S5–S9) revealed a gradual decrease in particle height from 1.2 nm (R‐CDs) to 1.0, 0.8, 0.6, and 0.4 nm (B‐CDs), respectively. The gradual decrease in the average thickness from R‐CDs to B‐CDs is presumably attributed to the exfoliation of the internal graphitized‐like structure of CDs, which results in the reduction in particle thickness [39]. Transmission electron microscopy (TEM) was employed to evaluate the morphology and lateral size distribution of the CDs (Figure 2h–l). All samples display uniform spherical morphology with excellent dispersibility. Size analysis from 50 randomly selected particles (see insets in Figure 2h–l) showed a clear size reduction trend: from 5.2 nm (R‐CDs) to 4.3, 3.9, 3.1, and 2.8 nm for O‐CDs to B‐CDs. This size‐reduction trend is further supported by dynamic light scattering (DLS) measurements (Figure S10), which further support the UV‐induced particle size reduction. High‐resolution TEM (HRTEM) images (Figure 2m‐q) revealed distinct lattice fringes in all samples, with an interplanar spacing of approximately 0.21 nm, corresponding to the (100) plane of graphitic carbon. In addition to the observed reduction in particle size, a gradual decrease in the clarity of the lattice fringes is evident along the transformation from R‐CDs to B‐CDs [40]. The progressive reduction in CD size and crystallinity under UV irradiation suggests a photoetching mechanism, which is further verified by the evolution of optical properties in the following section.

### 2.2 Optical Properties of CDs

**FIGURE 3:**
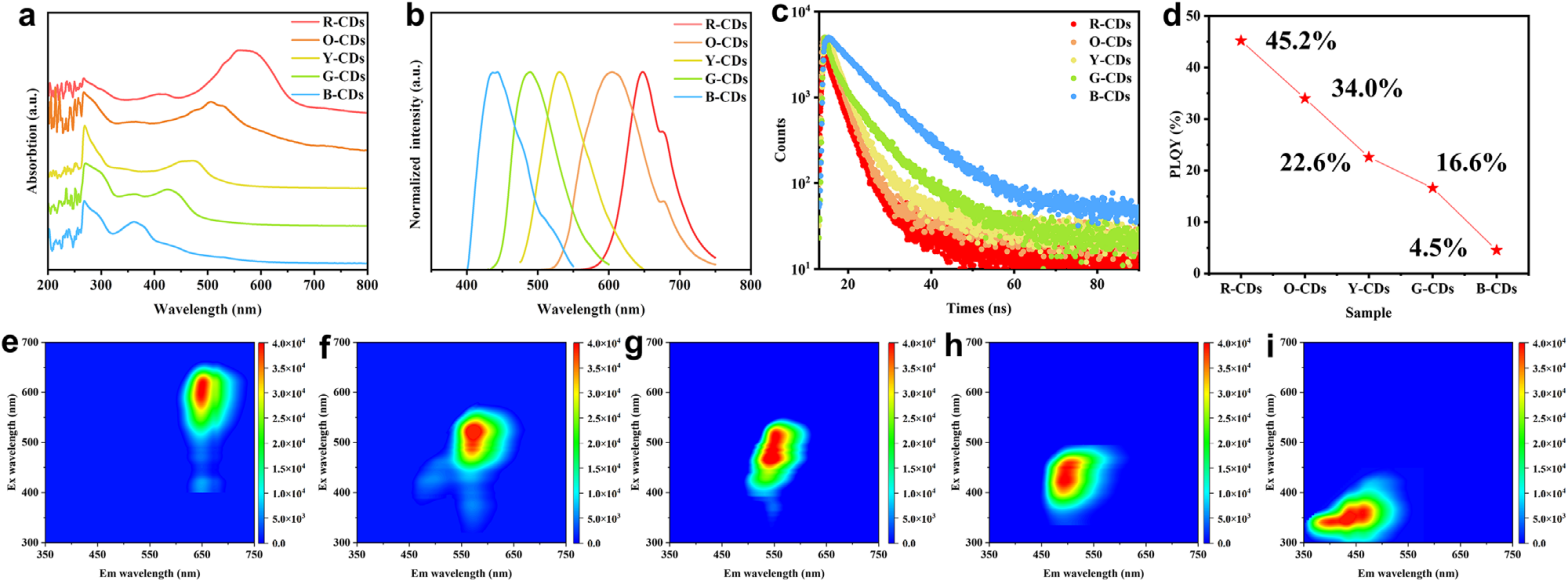
(a) UV–vis absorption spectra of multicolor CDs. (b) Normalized fluorescence emission spectra of multicolor CDs. (c) Time‐resolved PL decay of multicolor CDs. (d) The absolute PLQY of multicolor CDs. Fluorescence excitation‐emission maps of R‐CDs (e), O‐CDs (f), Y‐CDs (g), G‐CDs (h), and B‐CDs (i).

Multicolor CDs suspensions in DMF were separately prepared at a concentration of 0.01 mg/mL, and their optical properties were systematically characterized via ultraviolet–visible absorption and fluorescence spectroscopy. Figure 3a,b present the UV–vis absorption and photoluminescence (PL) spectra of multicolor CDs, both of which exhibit a pronounced blue shift with increasing irradiation time. As shown in Figure 3a, the disordered absorption bands below 260 nm are caused by the solvent. All samples exhibit two distinct absorption regions: a high‐energy band below 300 nm and a low‐energy band in the visible‐light region. The former, which remains nearly unchanged across the series, is attributed to π–π* transitions within the sp^2^‐conjugated carbon core [41]. In contrast, the visible‐range absorption peaks display distinct, color‐dependent shifts. Specifically, R‐CDs, O‐CDs, Y‐CDs, G‐CDs, and B‐CDs exhibit excitonic absorption bands centered at 570, 507, 471, 424, and 361 nm, respectively [14]. These peaks are associated with surface‐state transitions influenced by surface functional groups. The progressive blue shift of these excitonic features with increasing irradiation time indicates continuous alteration of the surface states and local chemical environments. Notably, as we can see in Figures S11–S15 the optimal excitation wavelengths of the CDs closely match their respective excitonic absorption maxima, suggesting a strong correlation between surface‐state absorption and excitation behavior [42]. Normalized fluorescence emission spectra reveal a similar trend in Figure 3b. The emission maxima gradually shift from 657 nm (R‐CDs) to 573, 530, 490, and finally 443 nm (B‐CDs), reflecting a tunable red‐to‐blue fluorescence evolution. Corresponding Commission Internationale de l’Éclairage (CIE) chromaticity coordinates (Figure S16) quantitatively confirm the full‐color emission range spanning red, orange, yellow, green, and blue. Moreover, R‐CDs, O‐CDs, Y‐CDs, and G‐CDs all exhibit excitation‐independent fluorescence behavior, as evidenced by their invariant emission peak positions under different excitation wavelengths (Figure 3e–h). This further confirms that the fluorescence emission originates from well‐defined band‐edge exciton transitions. In contrast, in Figure 3i, B‐CDs display a broader peak upon excitation with high‐energy light (λ < 345 nm). This phenomenon arises from mismatched energy levels and incomplete non‐radiative relaxation of the core state, thereby generating characteristic core‐state emission [43]. Figure S17 illustrates the excellent photoluminescence temporal stability of these CDs: almost no decrease in fluorescence intensity is observed after storage in powder form and aqueous dispersion for 10 days. The fluorescence quantum yields (QY) of the CDs were determined to be 45.2% (R‐CDs), 34.0% (O‐CDs), 22.6% (Y‐CDs), 16.6%(G‐CDs), and 4.5% (B‐CDs), as shown in Figure 3d. The quantum yield gradually decreases with the progressive blue shift, which is an inevitable consequence of the combined effects of photoetching‐induced surface defect generation and the destruction of highly efficient emissive centers. This behavior is consistent with the observed reduction in particle size and fluorescence blue shift: while photoetching leads to smaller CD sizes, it simultaneously introduces detrimental quenching defects that suppress radiative recombination [44]. Figure 3c shows the time‐resolved PL decay of multicolor CDs. The increasing lifetimes from R‐CDs (4.45 ns) to B‐CDs (8.54 ns) correlate with the increasing degree of UV‐induced structure degradation, further supporting the evolution of emission characteristics driven by surface and structural changes.

### 2.3 Chemical Characterizations of CDs

**FIGURE 4:**
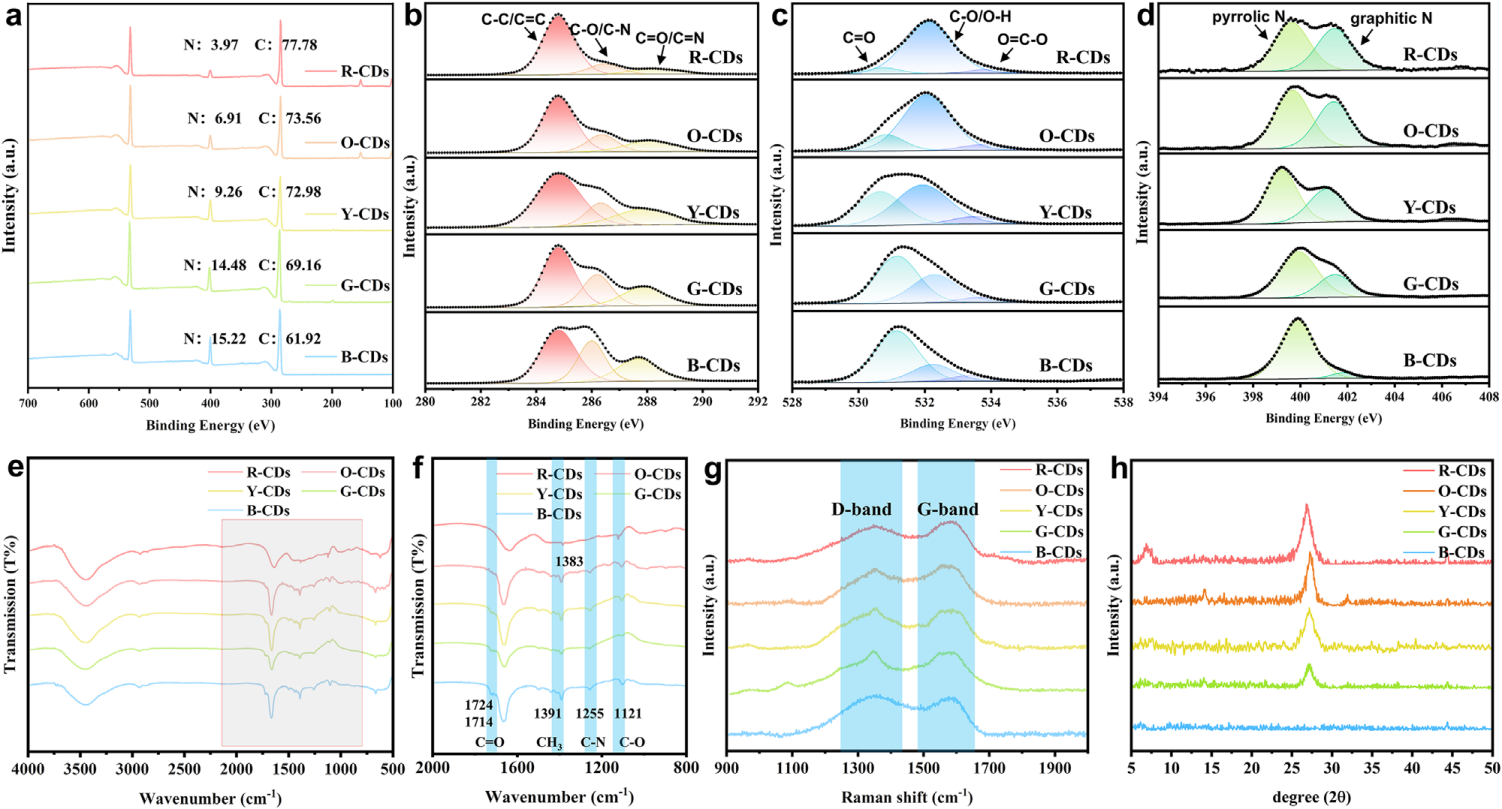
(a) XPS survey spectra of multicolor CDs. (b) High‐resolution C 1s spectra of multicolor CDs. (c) High‐resolution O 1s spectra of multicolor CDs. (d) High‐resolution N 1s spectra of multicolor CDs. (e‐f) The FTIR spectrum of multicolor CDs. (g) Raman spectrum of multicolor CDs. (h) The XRD spectrum of multicolor CDs.

The effects of UV irradiation on the chemical composition of the CDs were investigated using X‐ray photoelectron spectroscopy (XPS). As shown in Figure 4a, all multicolor CDs—R‐CDs, O‐CDs, Y‐CDs, G‐CDs, and B‐CDs—are composed of carbon (C), nitrogen (N), and oxygen (O). The atomic percentage of carbon gradually decreases from 77.8% to 73.56%, 72.98%, 69.16%, and 61.92%, while the nitrogen content increases from 3.97% to 6.91%, 9.26%, 14.48%, and 15.22%, respectively. This significant reduction in carbon content can be attributed to UV‐induced degradation of the carbon framework and the detachment of surface organic groups, which are further oxidized into volatile species and lost [45]. Consistent with the XPS, the total organic carbon (TOC) measurements (Figure S18) also displayed a gradual decline in carbon content, indicating continuous carbon depletion and supporting the proposed photoetching mechanism. A significant increase in nitrogen content was observed during the photoreaction. When the solvent was replaced with water and ethanol and the irradiation time was sufficiently prolonged, XPS spectra (Figure S19) showed that no comparable nitrogen enrichment appeared in the final products. This confirms that the increased nitrogen content arises from nitrogen‐containing active species generated by the decomposition of DMF. High‐resolution C 1s spectra (Figure 4b) can be fitted into three main peaks centered at 284.8, 286.4, and 287.9 eV, corresponding to C═C, C─N/C─O, and C═O/C═N, respectively [46]. As the CDs evolve from R‐CDs to B‐CDs, the proportion of C═C continuously decreases, while C─N/C─O and C═O/C═N components steadily increase. This trend suggests progressive destruction of graphitic domains and the introduction of nitrogen‐ and oxygen‐containing functional groups upon UV exposure. O 1s spectra (Figure 4c) display two peaks at 530.6 and 532.2 eV, attributed to carbonyl (C═O) and hydroxyl (─OH) groups, respectively [47]. During the transformation from R‐CDs to B‐CDs, the intensity of the C═O peak increases, while that of ─OH decreases, suggesting that hydroxyl groups are likely oxidized into carbonyl groups during UV irradiation. N 1s spectra (Figure 4d) reveal two peaks at 399.5 and 401.6 eV, corresponding to pyrrolic nitrogen and graphitic nitrogen, respectively [40]. Initially, nitrogen is mainly incorporated as graphitic N within the carbon core during solvothermal synthesis. However, extended UV exposure leads to the breakdown of the graphitic framework and the formation of edge‐localized pyrrolic N, resulting in an increased content of pyrrolic nitrogen. Fourier‐transform infrared spectroscopy (FTIR, Figure 4e) was employed to further examine the structural and surface functional changes. Compared to R‐CDs, the irradiated CDs (O‐, Y‐, G‐, and B‐CDs) show enhanced peaks at 1714 and 1724 cm^−1^, associated with C═O stretching. Meanwhile, the ─OH stretching band at 1121 cm^−1^ diminishes, confirming the conversion of hydroxyl to carbonyl groups [31]. A stronger peak at 1255 cm^−1^, attributed to C─N stretching, indicates higher pyrrolic nitrogen content and reduced aromaticity. Additionally, the enhanced signal at 1383 cm^−1^, with a slight shift to 1391 cm^−1^, is indicative of increased non‐conjugated sp^3^ carbon (e.g., ─CH_3_) and nitrogen‐containing moieties (e.g., N─CH_3_), which alter the local electronic structure [48]. These results corroborate the XPS findings, confirming the substitution of ─OH with C═O and progressive nitrogen doping, accompanied by disruption of sp^2^‐conjugated carbon domains. Raman spectra (Figure 4g) show characteristic D and G bands at approximately 1350 and 1583 cm^−1^, corresponding to disordered and graphitic sp^2^ carbon, respectively [49]. The I_D_/I_G_ ratio increases progressively from 0.81 to 0.88, 0.95, 1.04, and 1.12, indicating increased structural disorder and defect density with UV exposure. X‐ray diffraction (XRD, Figure 4h) was used to probe the crystalline structure. Distinct (002) diffraction peaks at ∼27° are observed for R‐, O‐, Y‐, and G‐CDs, corresponding to the interlayer spacing of ∼0.34 nm in graphitic carbon [50]. However, B‐CDs show no such peak, implying that prolonged UV‐induced etching and exfoliation produce monolayer graphene‐like domains lacking interlayer stacking. Under ultraviolet irradiation, the graphitic‐like layers of the CDs were progressively exfoliated and structurally disrupted. To verify this hypothesis, the CD–DMF solution was subjected to high‐frequency ultrasonication for different durations, with an ice–water bath employed to eliminate thermal effects. As the ultrasonication time increased, the fluorescence of the CDs exhibited a significant blue shift (Figure S20), and the fluorescence spectrum graph (Figure S21) revealed a gradual shift of the emission peak from 657 to 476 nm. The corresponding UV–vis absorption spectra (Figure S22) showed a continuous decrease in the long‐wavelength region accompanied by an increase in the short‐wavelength region, consistent with size reduction and partial exfoliation. Compared with UV irradiation, the blue shift induced by ultrasonication was less pronounced, reaching only 476 nm, with the fluorescence color changing to green. These observations indicate that, beyond the physical exfoliation of graphitic layers, ultraviolet irradiation also triggers surface chemical transformations, particularly the modification of functional groups, which collectively contribute to the observed continuous spectral evolution.

### 2.4 Luminescence Mechanism of CDs

**FIGURE 5:**
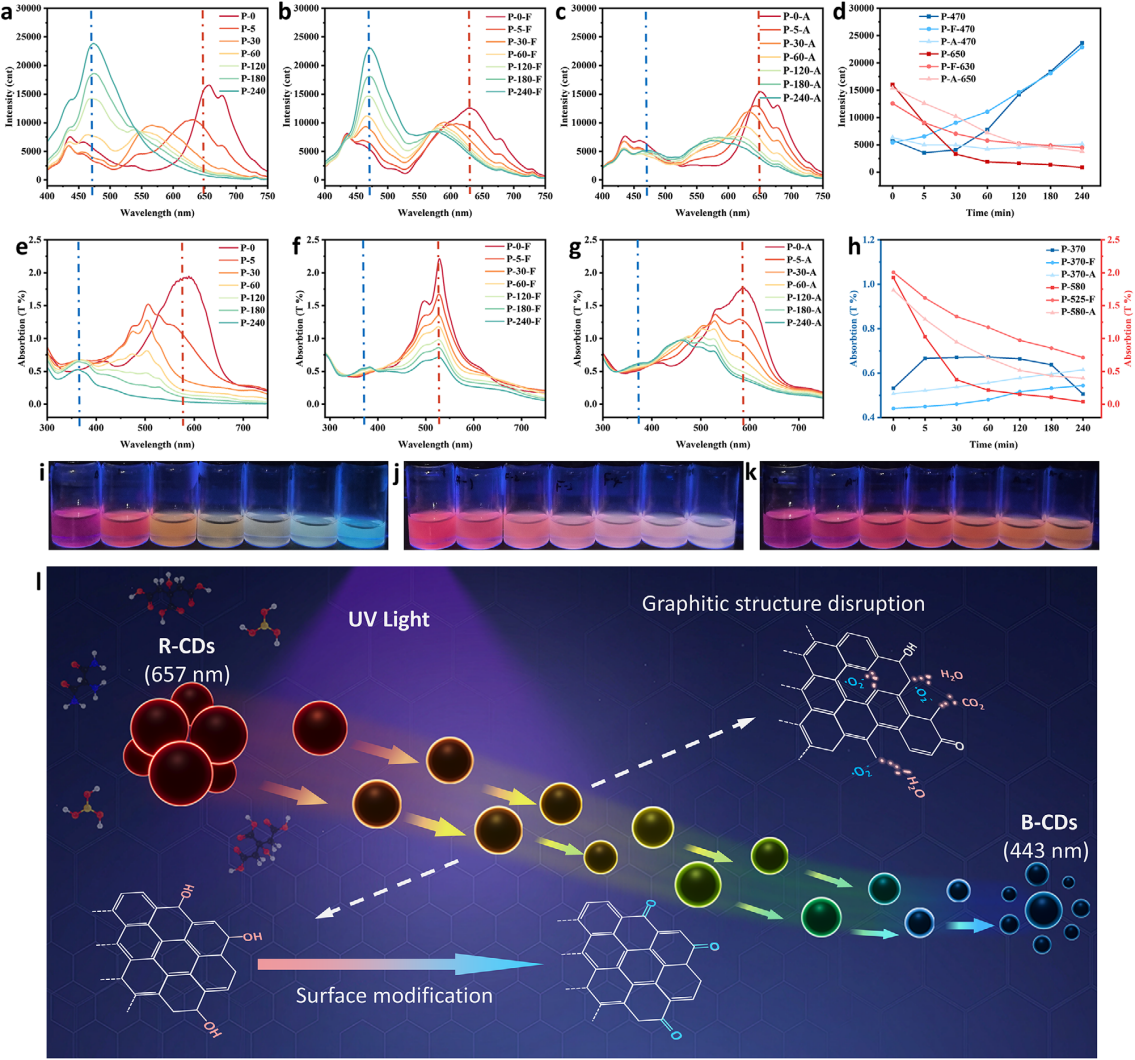
Fluorescence spectra of R‐CDs solutions (a), R‐CDs solutions containing TFA (b), and R‐CDs solutions containing AA (c) at varying UV exposure times. (d) Red and blue emission peak intensity evolution of CDs under various treatment conditions. UV–vis absorption spectra of R‐CDs solutions (e), R‐CDs solutions containing TFA (f), and R‐CDs solutions containing AA (g) at varying UV exposure times. (h) Absorption peak intensity evolution of R‐CDs at red and blue under various treatment conditions. Photographs (under 365 nm UV lamp) of R‐CDs solutions (i), R‐CDs solutions containing TFA (j), and R‐CDs solutions containing AA (k) at varying UV exposure times. (l)Proposed structural evolution mechanisms of R‐CDs under UV illumination.

Based on the above characterizations, it is inferred that ultraviolet irradiation not only induces etching and exfoliation of the CD structure but also alters the surface functional groups. Electron paramagnetic resonance (EPR) spectroscopy showed that the TEMPO radical signal was quenched upon UV irradiation of R‐CDs (Figure S23a), implying abundant unpaired electrons. This indicates that photoinduced radicals dominate the structural evolution of CDs. Using DMPO as a spin trap, a characteristic 1:1:1:1 quartet signal was observed under UV light (Figure S23b), verifying that superoxide radicals are the main species accounting for CD etching [51]. Superoxide radicals were detected continuously during irradiation; their signal gradually decreased with prolonged illumination and stabilized after 60 min (Figure S23c). To elucidate the factors influencing fluorescence evolution, we performed in situ absorption and fluorescence measurements on R‐CDs solutions under UV irradiation for 0–240 min. Parallel experiments were conducted on R‐CDs solutions containing trifluoroacetic acid (TFA) or ascorbic acid (AA) as additives. Figure 5a–c shows the fluorescence spectra of pristine R‐CDs (P‐0 to P‐240), TFA‐modified R‐CDs (P‐0‐F to P‐240‐F), and AA‐modified R‐CDs (P‐0‐A to P‐240‐A), respectively. All spectra were recorded under an excitation wavelength of 365 nm. Corresponding optical images under UV light are displayed in Figure 5i–k. The fluorescence evolution of three sample sets was compared in both the short‐wavelength (blue emission) and long‐wavelength (red emission) regions (Figure 5d). A distinct trend was observed: both trifluoroacetic acid (TFA) and ascorbic acid (AA) effectively suppressed the decrease in red‐emission intensity, with TFA exhibiting a stronger inhibitory effect. However, their influences diverged in the blue‐emission region. TFA showed almost no ability to suppress the enhancement of blue fluorescence, whereas AA, functioning as an efficient radical scavenger, nearly completely inhibited the growth of the blue‐emission peak. These results indicate that the suppression of the red‐shift attenuation mainly involves proton‐assisted stabilization, while the inhibition of blue fluorescence enhancement arises from radical‐quenching processes. UV–vis absorption spectra of the three sample types under different irradiation times are presented in Figure 5e–g. In the long‐wavelength absorption region, TFA addition preserves the absorption peak position, indicating stabilization of surface‐group‐dominated n–π* transitions. The observed gradual decrease in intensity is attributed to structural etching of the CDs. Conversely, AA does not prevent changes in this region; the absorption peak not only decreases but also blue‐shifts, suggesting substantial modification of surface states. In the short‐wavelength region, decreased surface‐state absorption can enhance the relative contribution of intrinsic‐state emission. However, structural defects introduced by etching may quench intrinsic fluorescence. The R‐CDs sample shows relatively stable short‐wavelength absorption, which only decreases significantly after prolonged UV exposure and degradation. TFA effectively suppresses surface state changes, whereas AA inhibits structural etching, both resulting in a slow increase of absorption upon irradiation. Based on the distinct variations observed in the fluorescence and UV spectra, the proposed mechanism for the structural evolution of the CDs upon UV irradiation is depicted in Figure 5l. Upon exposure to UV light, the R‐CDs undergo transformations in both their surface functionalities and graphitic carbon framework: hydroxyl‐rich groups are progressively oxidized to carbonyl moieties, while the internal carbon skeleton is etched, ultimately resulting in the exfoliation of the layers. In inhibition experiments, TFA supplies protons that suppress surface overoxidation through protonation, stabilizing long‐wavelength emissive centers, but cannot prevent photogenerated radicals from etching the sp^2^ carbon core. In contrast, AA acts as a radical scavenger, effectively inhibiting etching and exfoliation, it cannot fully protect hydroxyl groups from oxidation. In summary, the fluorescence color evolution of CDs arises from the combined effects of changes in both the carbon core and surface functional groups. Initially, hydroxyl‐to‐carbonyl conversion narrows the bandgap; subsequently, progressive destruction of the layered structure from outside inward leads to exfoliation. The newly exposed surfaces are further etched by photoinduced radicals, facilitating continuous surface group transformations until monolayer CDs are formed. To validate this mechanism, a control experiment was conducted by heating an R‐CDs solution at 100°C for 2 h in the dark, yielding a sample named CDs‐2h‐Dark. This sample exhibited orange fluorescence similar to O‐CDs (Figure S24), indicating that the red‐to‐orange shift can be attributed solely to surface functional group transformation without involvement of light‐induced radicals, consistent with our proposed pathway. Extending heating to 12 h (CDs‐12 h‐Dark) did not induce further fluorescence shifts (Figure S25), suggesting that in the absence of light, the carbon core remains intact and fluorescence variation ceases once reactive surface sites are consumed. Furthermore, although all multicolor CDs share an n–π* surface‐state‐dominated emission mechanism, the blue‐emissive CDs formed after prolonged irradiation differ from those produced at early stages. This was confirmed by pH sensitivity tests of four CDs and B‐CDs (Figures S26 and S27). Four CDs exhibit pronounced pH‐dependent fluorescence shifts due to protonation/deprotonation of surface groups, affecting surface charge and emission wavelength. In contrast, B‐CDs show negligible emission shifts with pH changes, indicating fluorescence originates from more stable and inert surface states.

**FIGURE 6:**
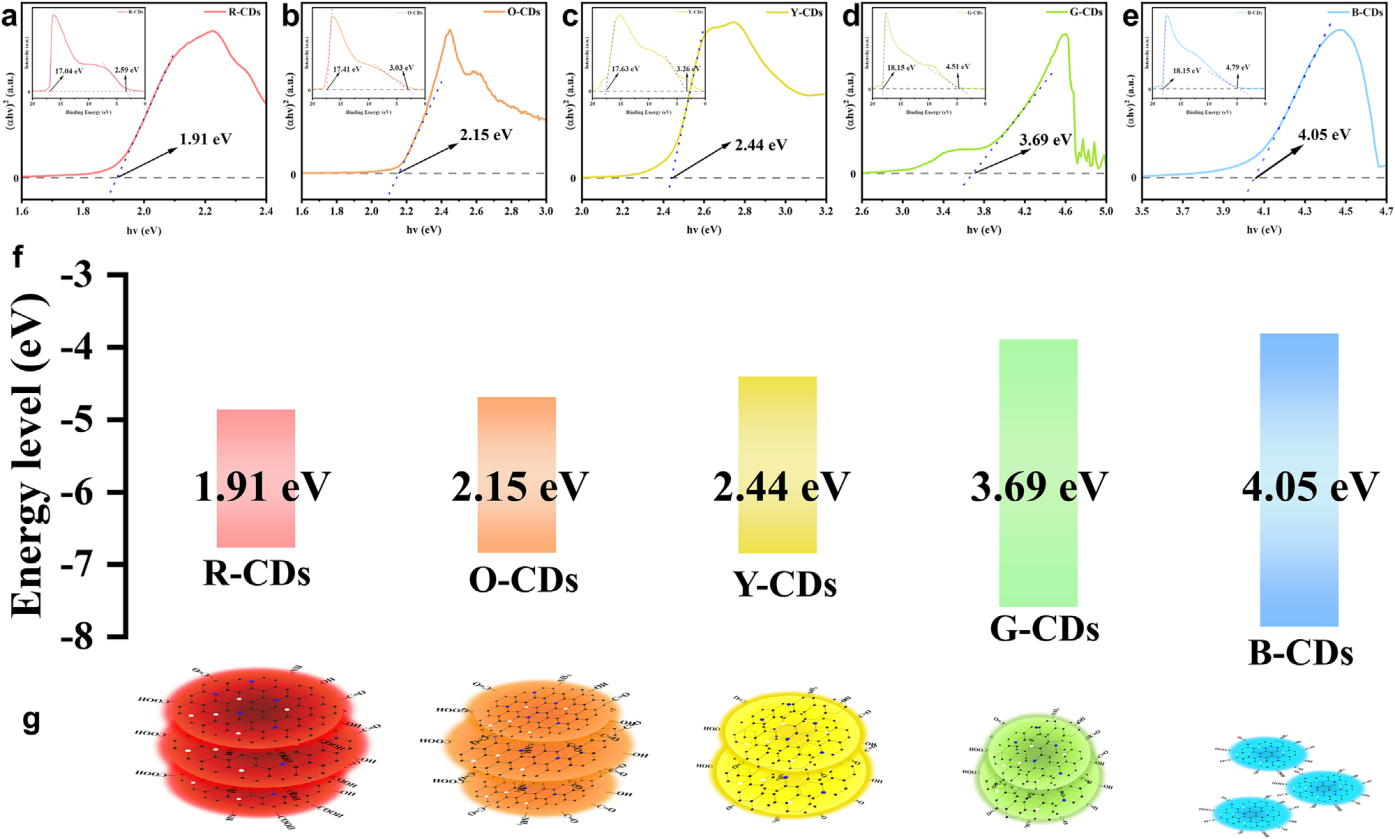
(a–e) (αhν)^2^ versus hν curves and UPS spectra (Inset) of R‐CDs, O‐CDs, Y‐CDs, G‐CDs, and B‐CDs. (f) HOMO/LUMO energy levels and bandgaps of multicolor CDs. (g) Schematic structural models of multicolor CDs.

According to the UV–Vis absorption spectra shown in Figure 3a, the absorption edge (λ_edge_) of the multicolor CDs exhibits a progressive blue shift. Tauc plots of (αhν)^2^ versus hν were constructed based on the Kubelka–Munk theory, as shown in Figure 6a–e, and the optical band gaps were estimated using the tangent method. The calculated band gaps of R‐CDs, O‐CDs, Y‐CDs, G‐CDs, and B‐CDs were 1.91, 2.15, 2.44, 3.69, and 4.05 eV, respectively, indicating a gradual widening of the band gap upon UV irradiation. Furthermore, ultraviolet photoelectron spectroscopy (UPS) was performed to investigate the electronic structures of the CDs (insets of Figure 6a–e). The valence band maximum (HOMO level) was determined using the equation: E_VB_ = −[21.22 − (E_fermi_ − E_end_)], yielding HOMO energy levels of −6.77, −6.84, −6.85, −7.58, and −7.86 eV for R‐CDs, O‐CDs, Y‐CDs, G‐CDs, and B‐CDs, respectively. The corresponding conduction band minima (LUMO levels) were subsequently calculated by combining these HOMO values with the optical band gaps, resulting in LUMO levels of −4.86, −4.69, −4.41, −3.89, and −3.81 eV, respectively. As illustrated in Figure 6f, the observed downshift of the HOMO levels and upshift of the LUMO levels, in conjunction with the widened bandgap, are consistent with the effects of quantum confinement, decreased graphitization, and altered surface functional groups. Therefore, the observed blue shift in the fluorescence emission of the CDs can be attributed to the synergistic contributions of quantum size effects, bandgap modulation, and surface electronic structure variations. A schematic model illustrating the proposed multicolor CDs structure is provided in Figure 6g.

### 2.5 CDs‐Based LEDs

**FIGURE 7:**
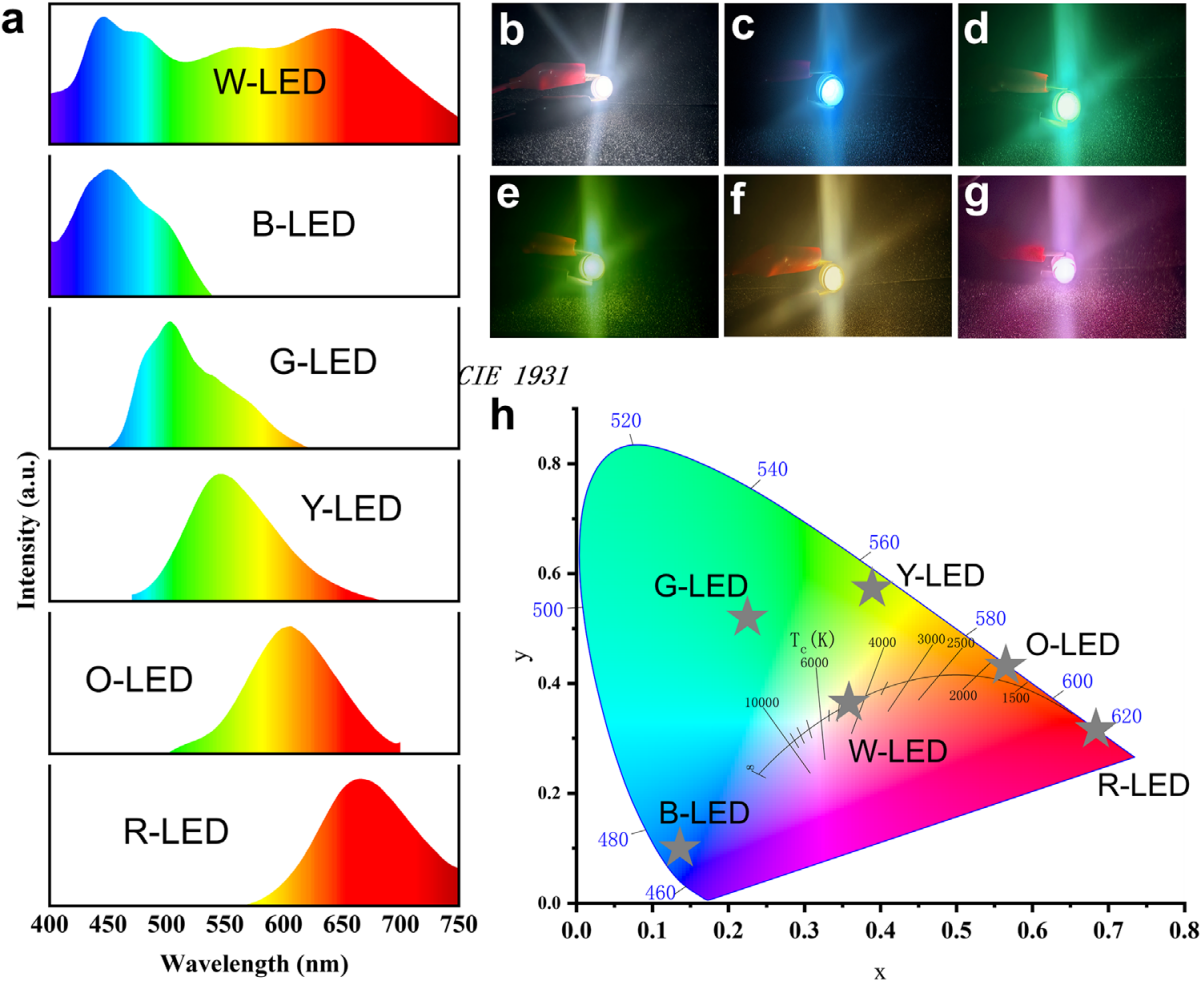
(a) Emission Spectra of Multicolor LEDs and W‐LEDs Under 50 mA Driving Current. Photographs of W‐LED (b), B‐LED (c), G‐LED (d), Y‐LED (e), O‐LED (f), and R‐LED (g) Operating at 3.5 V. (h) CIE coordinates of Multicolor LEDs and W‐LEDs.

The multicolor CDs prepared via the UV photoetching method were mixed with melamine resin to obtain multicolor fluorescent powders. A GaN chip emitting at 365 nm was employed as the excitation source, and the multicolor CDs fluorescent powders were encapsulated onto the chip using epoxy resin to fabricate B‐LED, G‐LED, Y‐LED, O‐LED, and R‐LED. In addition, a white LED (W‐LED) was prepared using a mixed multicolor CDs fluorescent powder. The photographs and structural schematic diagrams of the fabricated LEDs are shown in Figure S29. All LEDs were operated at a voltage of 3.5 V and a driving current of 50 mA. The electroluminescence photographs of the various LEDs under these operating conditions are shown in Figure 7b–g, and the corresponding emission spectra are presented in Figure 7a. The emission spectra of the multicolor LEDs closely resemble the photoluminescence spectra of the corresponding multicolor CDs. For the white LED, the emission spectrum spans the entire visible region from red to blue. The CIE chromaticity coordinates are (0.36, 0.36), and a high color rendering index (CRI) of 92 is achieved (Figure 7h). This represents a significant improvement compared with the CRI of 81 for commercially available white LEDs (Figure S30). Moreover, the correlated color temperature (CCT) is reduced to 4729 K. Thermogravimetric curves of the multicolor CDs reveal their high thermal stability (Figure S28a), which enables the W‐LED to maintain nearly unchanged spectra under high‐temperature conditions (Figure S28b). Upon continuous operation for 12 h, the reduction in Ra and the increase in CCT were both less than 10%, demonstrating their great application potential in high‐color‐rendering solid‐state lighting (Figure S31). Nevertheless, owing to the non‐overlapping optimal absorption regions of the multicolor CDs, the WLED devices fabricated through this strategy exhibit certain deficiencies in luminous efficiency, which only reaches 11 lm/W. We will optimize the device performance by designing rational configurations in our follow‐up work to enhance the efficiency.

### 2.6 CDs‐Based Multilevel Encryption System

**FIGURE 8:**
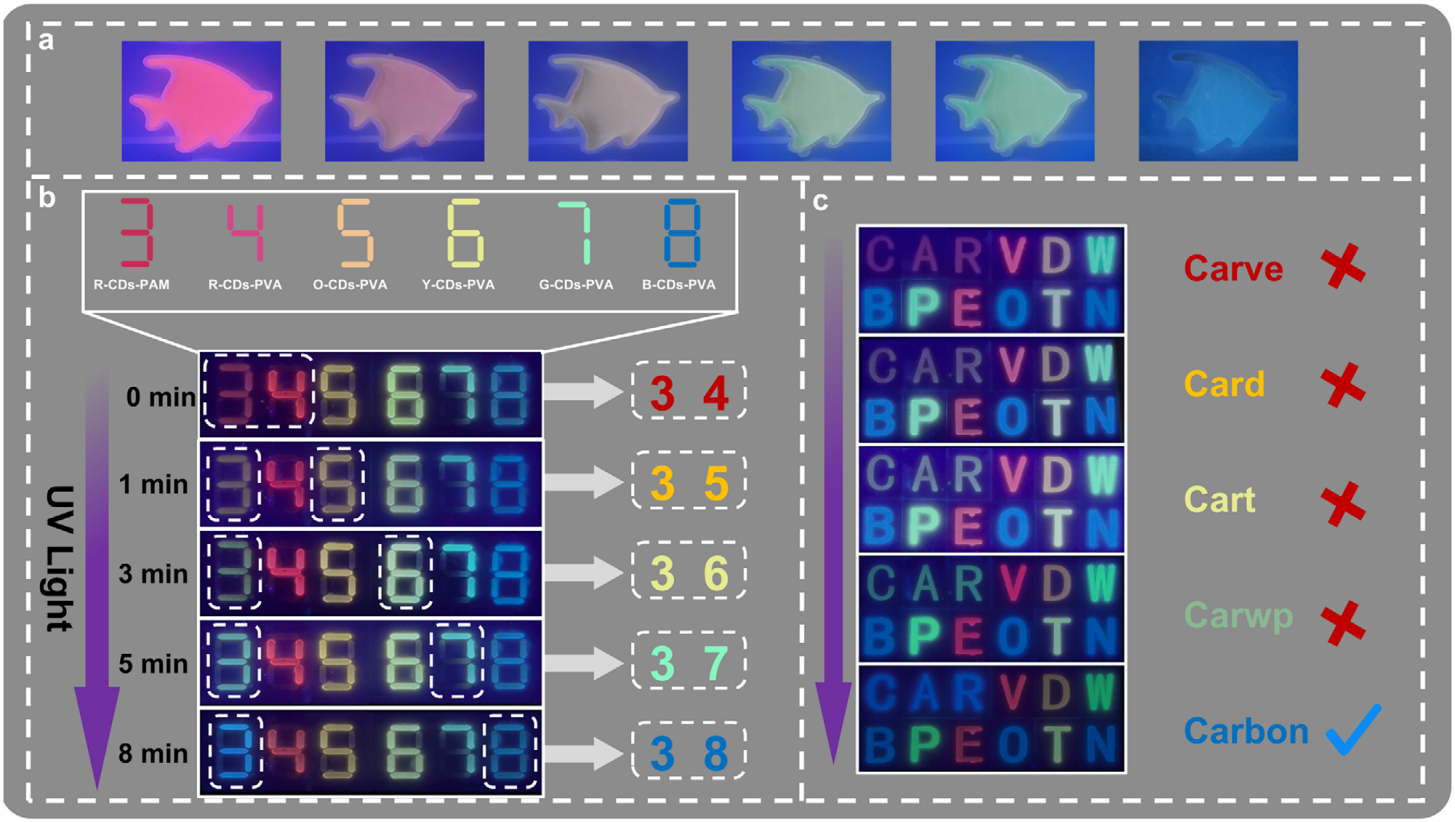
(a) Fluorescence images of R‐CDs‐PAM gel under UV lamp after exposure to mercury lamp irradiation for different durations. (b, c) Schematic diagram of the information encryption system based on R‐CDs‐PAM and multicolor CDs‐PVA gels.

To further explore the continuously tunable fluorescence properties of R‐CDs and expand their applicability in advanced functional materials, R‐CDs were embedded into a polyacrylamide (PAM) hydrogel matrix. The resulting R‐CDs‐PAM hydrogel exhibited red emission analogous to that of the original R‐CDs in solution, with negligible spectral deviation (Figure S32), indicating that the intrinsic optical properties of R‐CDs were well retained within the polymeric network. This result highlights the potential of incorporating R‐CDs into solid‐state polymer systems. Upon UV irradiation, the R‐CDs‐PAM hydrogel exhibited a continuous fluorescence evolution from red to orange, yellow, green, and eventually to blue, similar to the behavior observed in solution (Figure 8a). This successful implementation of dynamic fluorescence modulation in a solid‐state platform lays a foundation for the development of CD‐based information encryption technologies. At present, most CD‐based encryption systems rely on dual‐mode emission (fluorescence and room‐temperature phosphorescence) or require external stimuli such as temperature, pressure, or pH to achieve multilevel encoding, which significantly limits their information storage capacity [52, 53]. In this work, we addressed these limitations by employing the as‐prepared multicolor CDs (R‐CDs, O‐CDs, Y‐CDs, G‐CDs, and B‐CDs) and embedding them into polyvinyl alcohol (PVA) hydrogels along with ascorbic acid to stabilize their fluorescence. This yielded five stable multicolor hydrogels, designated as R‐PVA‐A, O‐PVA‐A, Y‐PVA‐A, G‐PVA‐A, and B‐PVA‐A (Figure S33). Leveraging the photoresponsive R‐CDs‐PAM gel and the stable multicolor PVA gels, a multilevel digital information encryption system was constructed, as illustrated in Figure 8b. In this system, each digit of the encoded message was represented by a distinct gel type. Initially, under 365 nm UV illumination, the unirradiated gels displayed digits “3” and “4” with similar red fluorescence, yielding a visible output of “34.” As UV irradiation time increased, the emission color of the R‐CDs‐PAM gel progressively changed, enabling it to match the fluorescence color of different PVA reference gels. For instance, after 1 min of exposure, digits “3” and “5” exhibited similar orange fluorescence, resulting in an output of “35.” Further irradiation durations of 3, 5, and 8 min yielded outputs of “36,” “37,” and “38,” respectively, based on matching emission colors. Employing an analogous design principle, we constructed an anti‐counterfeiting platform featuring hierarchical information disclosure in Figure 8c. The genuine word “CARBON” is encrypted within a disordered letter matrix. Upon irradiation, a time‐dependent dynamic process is triggered: a series of spurious words sequentially manifests to create interference, ultimately culminating in the stable and unambiguous appearance of the true message after a prescribed exposure time. This strategy enabled up to five levels of dynamic encoding, allowing the encrypted information to be selectively retrieved at specific time intervals. Although the encryption system cannot be reused due to the irreversible etching characteristic of CDs, our five‐level encryption system exhibits significantly higher information storage capacity and better security compared with traditional dual‐mode encryption systems (fluorescence/room‐temperature phosphorescence). This system demonstrates a novel and highly secure information encryption strategy based on the tunable optical properties of CDs.

## 3 Conclusion

In this work, we present a UV irradiation‐mediated photoetching approach that enables continuous and precise fluorescence tuning of CDs within a single system, achieving full‐spectrum emission coverage from red (657 nm) to blue (443 nm). Systematic characterization (AFM/TEM) confirmed the progressive reduction in CD size under UV exposure. The fluorescence modulation mechanism was elucidated through carefully designed inhibition experiments using trifluoroacetic acid and ascorbic acid, which revealed the synergistic interplay between radical‐induced carbon core etching and solvent‐driven surface modification. The photoetching process involves gradual exfoliation of graphitic domains while surface hydroxyl groups undergo conversion to carbonyl groups, collectively modifying both surface state emission and quantum confinement effects to produce the characteristic blue‐shifting fluorescence. Critically, the emission wavelength can be precisely controlled solely by irradiation time, eliminating the need for complex multi‐step syntheses. Multicolor CDs were dispersed in melamine resin to obtain multicolor solid‐state phosphors, and multicolor LEDs and WLEDs with high color rendering index were successfully fabricated. The universality of this strategy was further demonstrated by integrating UV‐responsive CDs into polyacrylamide (PAM) hydrogels, which retained identical color‐switching behavior. This capability was leveraged to design a five‐level information encryption system – the highest achieved for CD‐based platforms to date – surpassing conventional dual‐mode encryption approaches. Our findings provide a simple yet powerful paradigm for the on‐demand customization of CD optical properties, while opening new avenues for their application in advanced anti‐counterfeiting technologies and multifunctional optoelectronics. The proposed photoetching mechanism may also inspire similar precision‐engineering strategies for other nanoscale carbon materials.

## 4 Methods

### 4.1 Materials

All reagents were of analytical grade and used without further purification. Citric acid, 2‐aminomalonamide, acrylamide (AM), N, N’‐methylenebisacrylamide (BIS), and PVA were purchased from Aladdin Industrial Corporation (Shanghai, China). Boric acid, Ammonium persulfate (APS), hydrochloric acid, sodium hydroxide, dimethylsulfoxide (DMSO), and N, N‐dimethylformamide (DMF) were obtained from Sinopharm Chemical Reagent Co., Ltd. (China).

### 4.2 Synthesis of R‐CDs

Citric acid (2 g), 2‐aminomalonamide (1 g), and boric acid (2 g) were dissolved in 25 mL of dimethylformamide (DMF) and subjected to solvothermal treatment at 180°C for 12 h. Upon completion, the reaction mixture was cooled to room temperature. The resulting solution was dialyzed against deionized water using a dialysis bag with a molecular weight cutoff (MWCO) of 1000 Da to remove small molecular impurities. The obtained solution was purified by silica column chromatography using a mixture of methanol and dichloromethane as the eluent. Finally, the R‐CDs solution was obtained.

### 4.3 Preparation of O‐CDs, Y‐CDs, G‐CDs, and B‐CDs

Thirty milligrams of R‐CDs were dissolved in 1 L of DMF and irradiated under a high‐pressure mercury lamp (Figure S1). The solutions collected after reaction times of 5, 15, 30, and 60 min were purified via silica gel column chromatography using a mixture of methanol and dichloromethane as the eluent, yielding O‐CDs, Y‐CDs, G‐CDs, and B‐CDs, respectively.

### 4.4 Preparation of R‐CDs‐PAM Hydrogel

A total of 29.22 g of acrylamide, 0.78 g of N, N’‐methylenebisacrylamide, and 8 mg R‐CDs were completely dissolved in 100 mL of deionized water under magnetic stirring. APS was then added as the initiator. The homogeneous precursor solution was transferred into a pre‐prepared gel mold using a pipette and allowed to stand at room temperature for 1 h to form the R‐CDs–polyacrylamide R‐CDs‐PAM hydrogel.

### 4.5 Preparation of Multicolor‐CDs‐PVA Hydrogel

Ten grams of PVA and 5 mg of multicolor CDs were dissolved in 100 mL of deionized water under stirring until a clear solution was obtained. The solution was then poured into a mold, followed by covering its surface with 20 mL of 5 wt.% boric acid solution. After standing for 1 h at room temperature, the multicolor‐CDs‐PVA hydrogel was successfully formed.

### 4.6 Characterization

The morphological analysis of the C‐CDs and B‐CDs nanostructures was conducted using a FEI Talos F200X G2 transmission electron microscope (TEM). Changes in the absorbance of the samples were measured with a Thermo Fisher Evolution 201 UV–vis spectrophotometer. X‐ray photoelectron spectroscopy (XPS) was performed on a Thermo Scientific K‐Alpha spectrometer equipped with an Al K‐alpha monochromatic X‐ray source and a pass energy of 150 eV. Fourier‐transform infrared spectroscopy (FTIR) was conducted using a Nicolet iS50 spectrometer to examine the molecular structures and bonding characteristics of the samples. Raman spectra were obtained using a Renishaw inVia spectrophotometer with 532 nm excitation. Fluorescence spectra, quantum yield (QY), and lifetime measurements were recorded with a Thermo Fisher Lumina fluorescence spectrophotometer and an Edinburgh Instruments FLS1000‐DD‐stm fluorescence spectrophotometer, the latter equipped with a calibrated integrating sphere. X‐ray diffraction (XRD) patterns were collected using a Rigaku MiniFlex600 diffractometer for lattice structure analysis of the samples. Ultraviolet photoelectron spectroscopy (UPS) measurements were conducted on a Thermo Fisher Scientific Nexsa spectrometer with a He I gas discharge lamp (hν  =  21.22 eV). Electron paramagnetic resonance (EPR) spectra of radicals and ‐O2‐ trapped by 5, 5‐dimethyl‐1‐pyrroline‐N‐oxide (DMPO) and 2, 2, 6, 6‐tetra‐methylpiperidine (TEMP), were recorded using an EPR spectrometer (EPR200‐Plus, CIQTEK Co., Ltd., China).

## Funding

This work was supported by the Graphene Powder & Composite Materials Research Center of Fujian Province (2017H2001) and The Xiamen Key Laboratory of Polymers & Electronic Materials (2024‐01).

## Conflicts of Interest

The authors declares no conflict of interest.

## Supporting information




**Supporting File**:advs75625‐sup‐0001‐SuppMat.docx.

## Data Availability

The data that support the findings of this study are available from the corresponding author upon reasonable request.
