# Supplementary material for: Fluorescence Tuning of Carbon Dots from Red to Blue via UV‐Induced Photochemical Etching
Source: Adv Sci (Weinh). 2026 May 8;13(43):e75625. doi: 10.1002/advs.75625 (PMC13336077; doi:10.1002/advs.75625)
Supplement: Supplementary file 1 — Supporting File:advs75625‐sup‐0001‐SuppMat.docx. [file ADVS-13-e75625-s001.docx]

Supporting Information

**Fluorescence Tuning of Carbon Dots from Red to Blue via UV-induced photochemical etching**

Nanzhi Zheng, Yuchen Yang, Jingwei Xu, Jincheng Gan, Yinuo An, Xinyu Li and Guohua Chen*

| **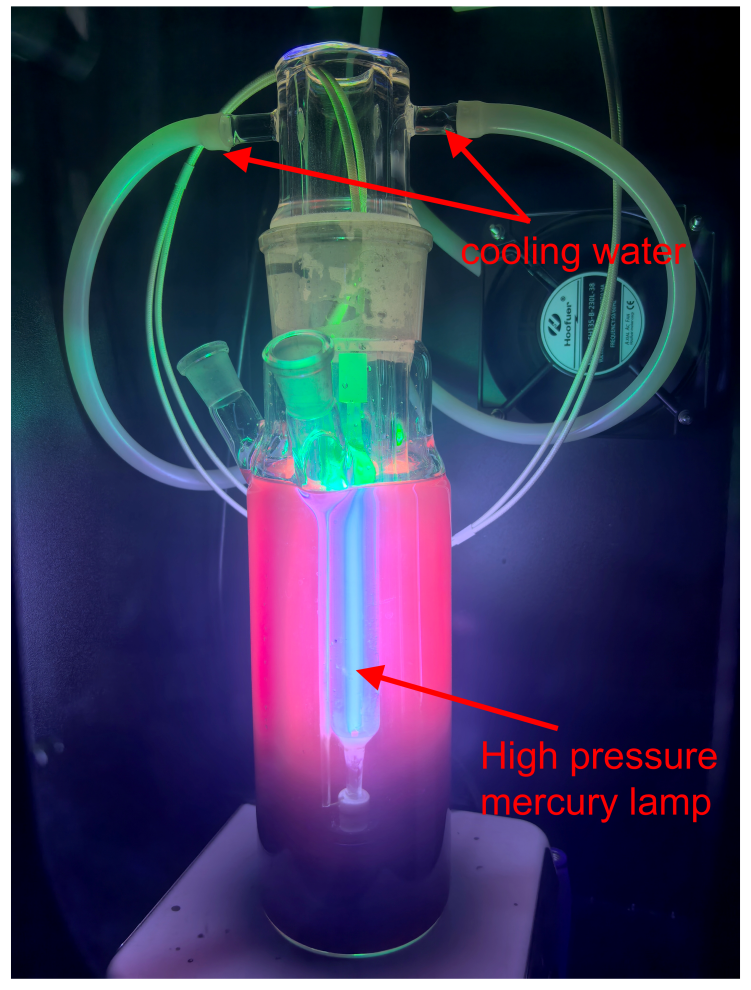** |
| --- |
| Figure S1. Operational state of the photoreaction and images of the experimental setup. |

| 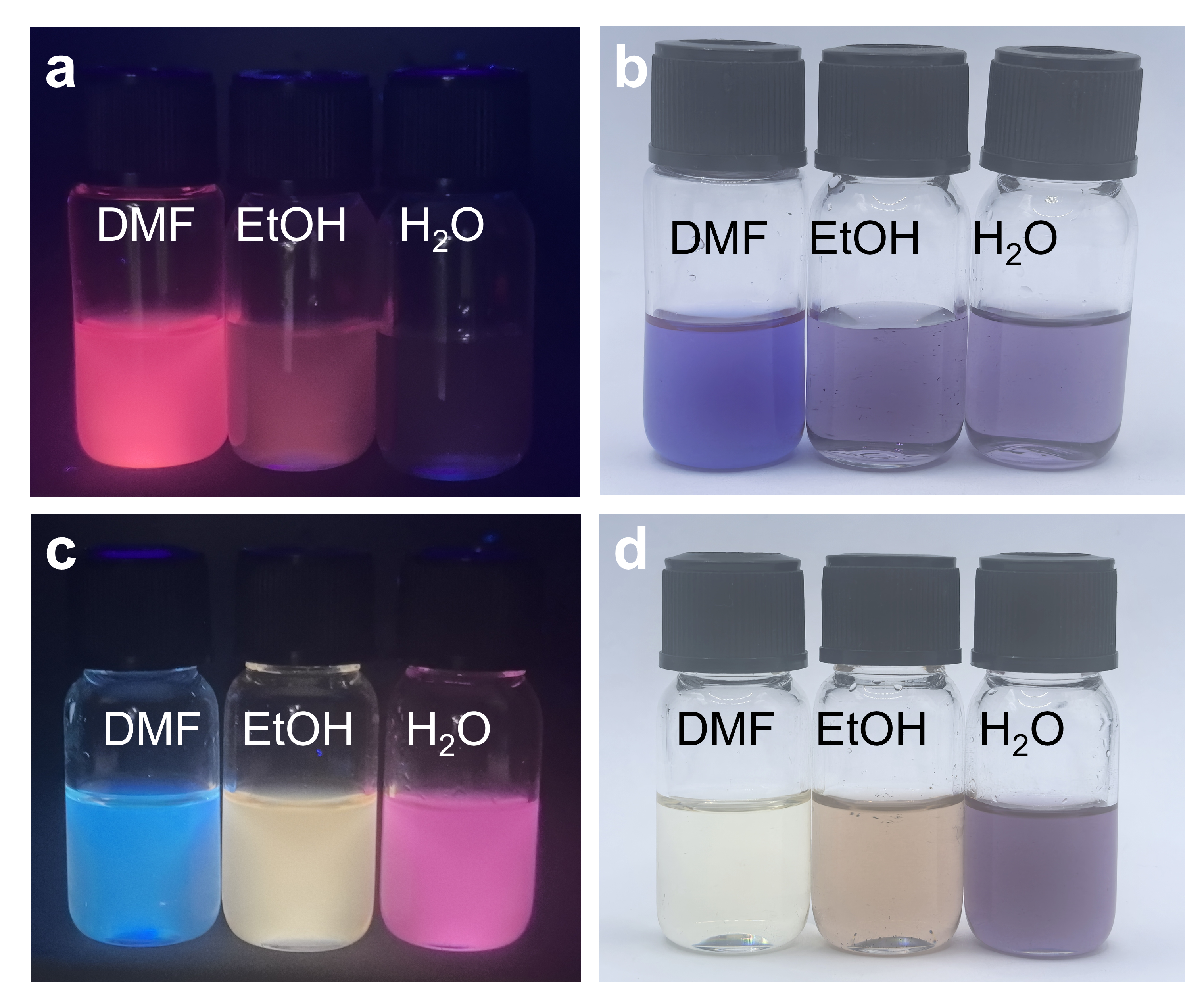 |
| --- |
| Figure S2. (a) Photographs of R-CDs in different solvents under UV irradiation; (b) dispersion states of R-CDs in various solvents; (c) photographs of R-CDs redispersed in DMF after 2 h of photoreaction in different solvents; (c) corresponding images under room light. |

| 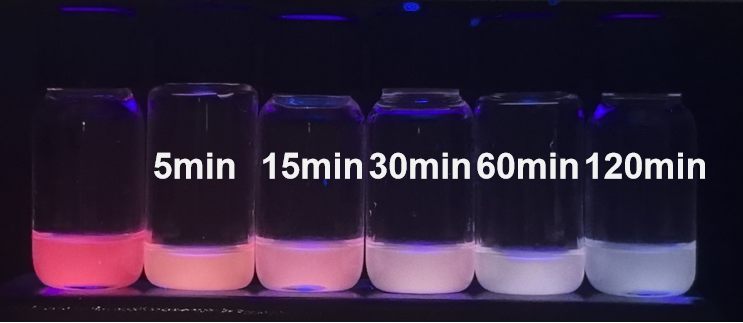 |
| --- |
| Figure S3. Photographs of 0.1 mg mL⁻¹ R-CDs in DMF under UV irradiation after different irradiation times. |

|  |
| --- |
| Figure S4. Normalized fluorescence spectra of R-CDs at different photoreaction times. |

| 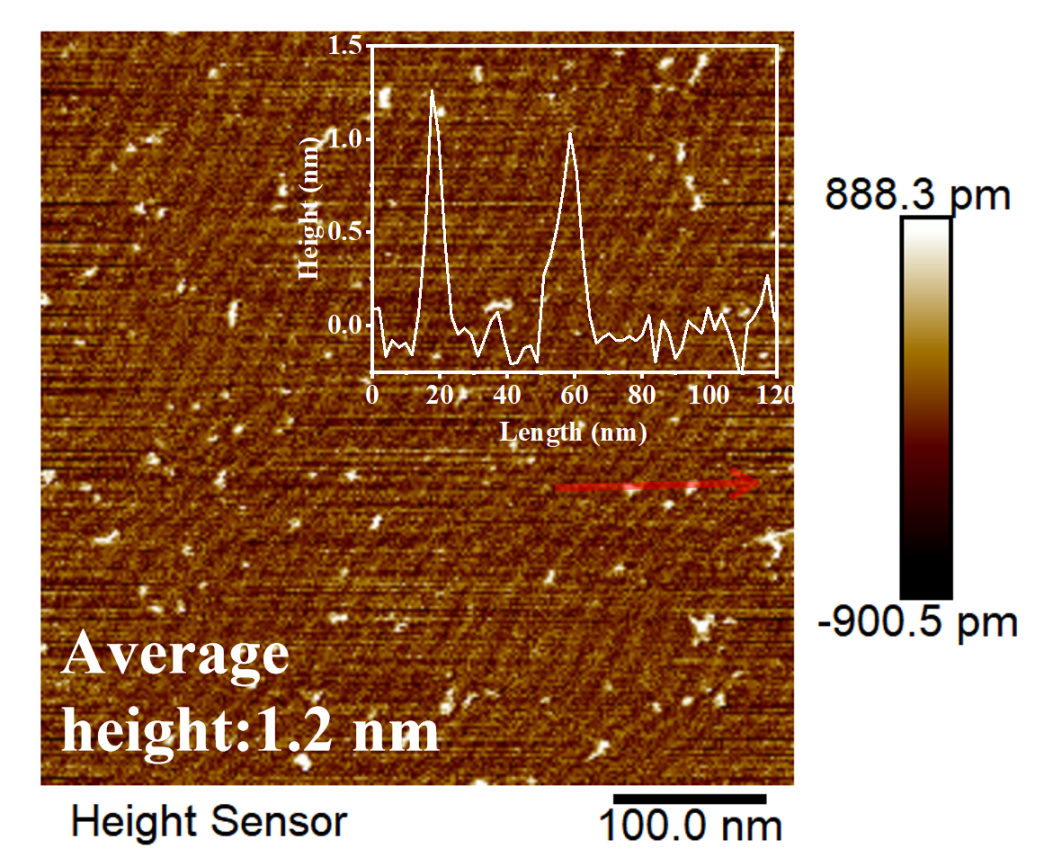 |
| --- |
| Figure S5. AFM images of R-CDs and height curves of the selected regions. |

| 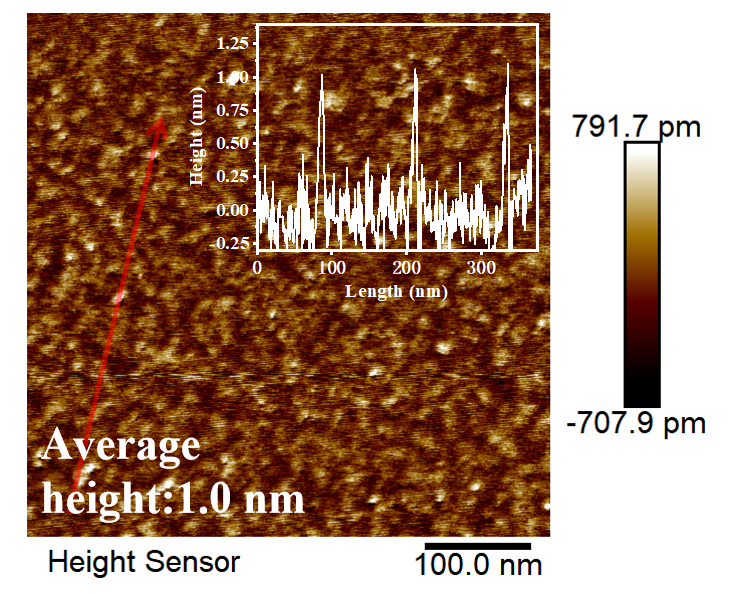 |
| --- |
| Figure S6. AFM images of O-CDs and height curves of the selected regions. |

| 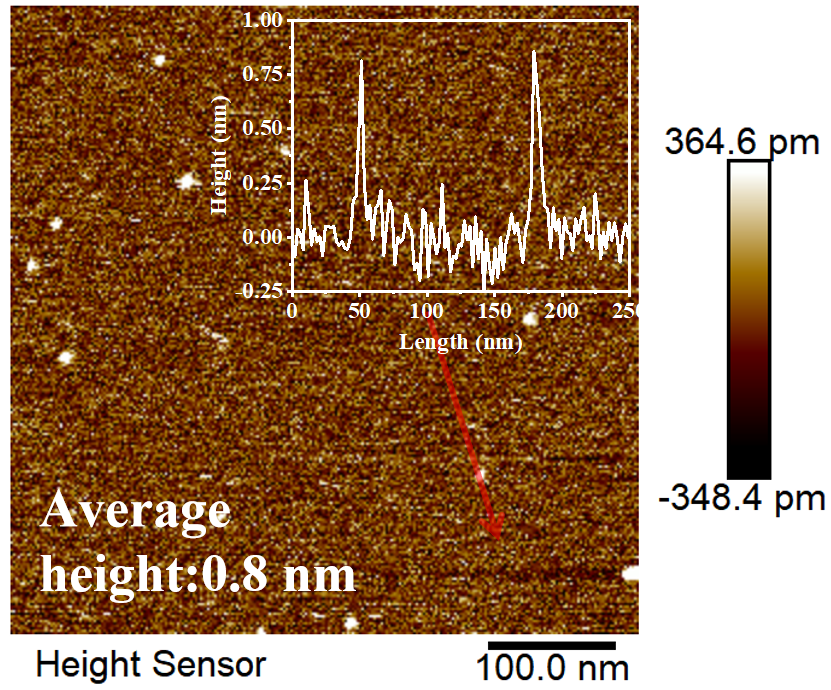 |
| --- |
| Figure S7. AFM images of Y-CDs and height curves of the selected regions. |

| 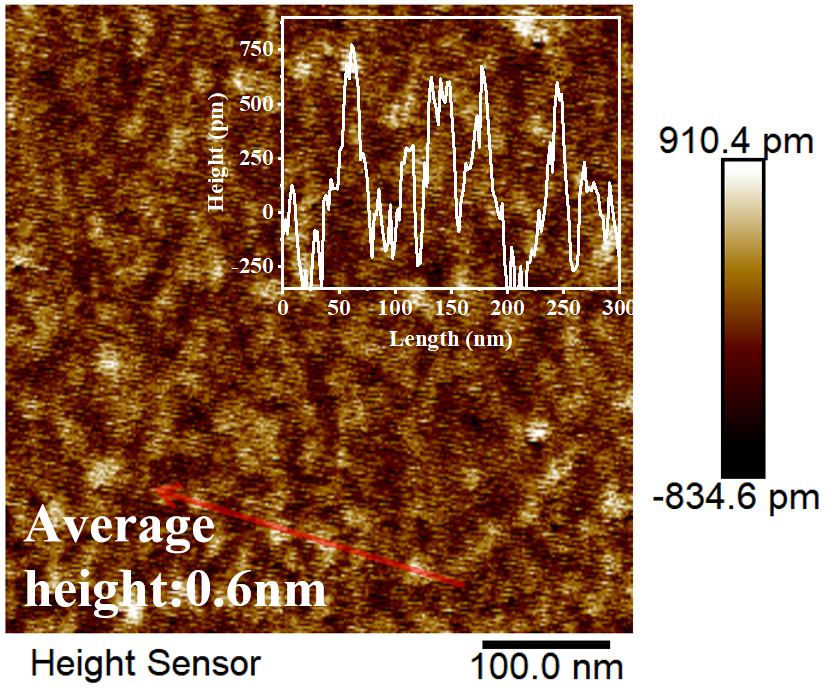 |
| --- |
| Figure S8. AFM images of G-CDs and height curves of the selected regions. |

| 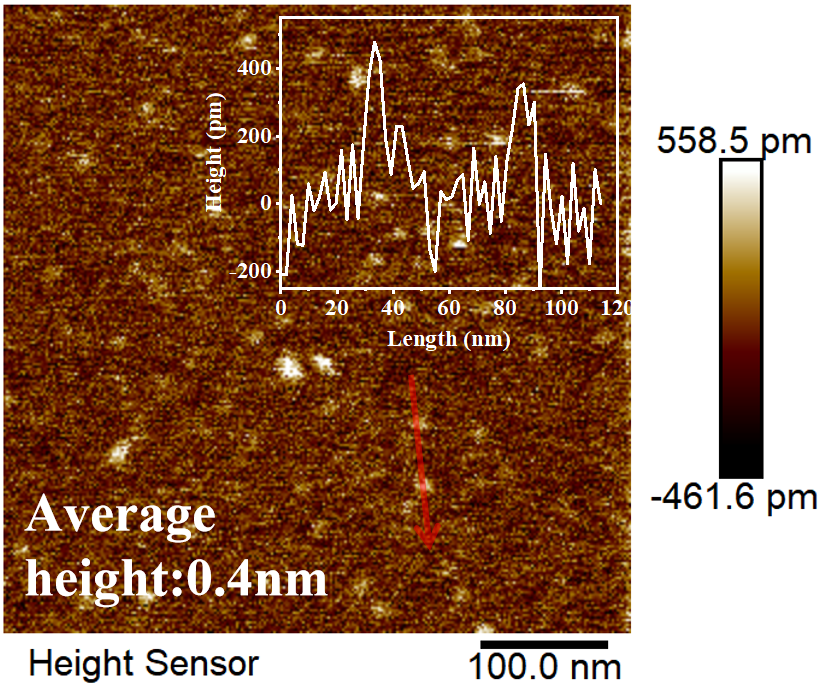 |
| --- |
| Figure S9. AFM images of B-CDs and height curves of the selected regions. |

|  |
| --- |
| Figure S10. Size diagram of multicolor CDs measured by dynamic light scattering. |

|  |
| --- |
| Figure S11. UV absorption spectrum, optimal fluorescence excitation spectrum and optimal fluorescence emission spectrum of R-CDs |

|  |
| --- |
| Figure S12. UV absorption spectrum, optimal fluorescence excitation spectrum and optimal fluorescence emission spectrum of O-CDs |

|  |
| --- |
| Figure S13. UV absorption spectrum, optimal fluorescence excitation spectrum and optimal fluorescence emission spectrum of Y-CDs |

|  |
| --- |
| Figure S14. UV absorption spectrum, optimal fluorescence excitation spectrum and optimal fluorescence emission spectrum of G-CDs |

|  |
| --- |
| Figure S15. UV absorption spectrum, optimal fluorescence excitation spectrum and optimal fluorescence emission spectrum of B-CDs |

| **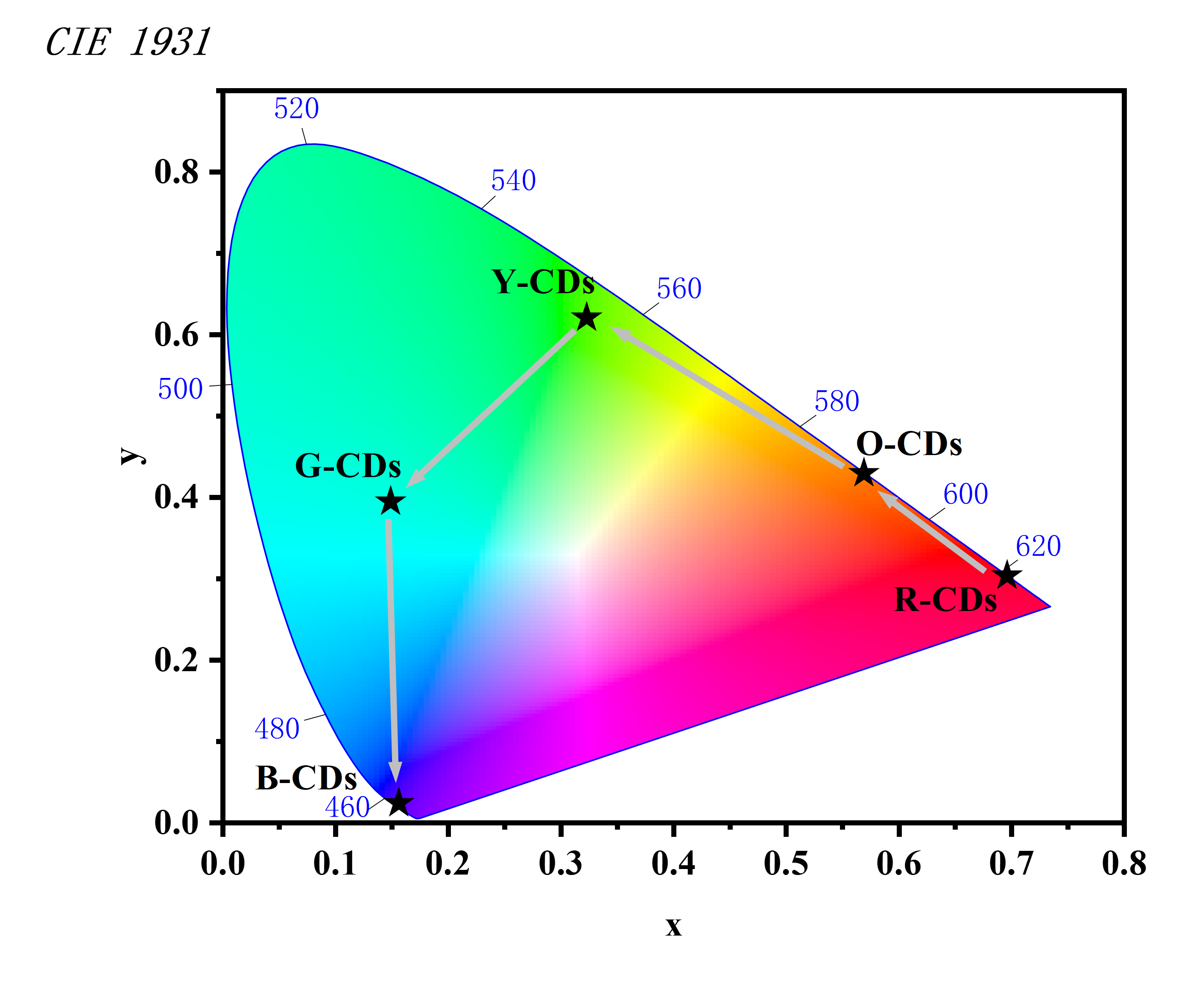** |
| --- |
| Figure S16. CIE chromaticity coordinate diagram of multicolor CDs. |

| 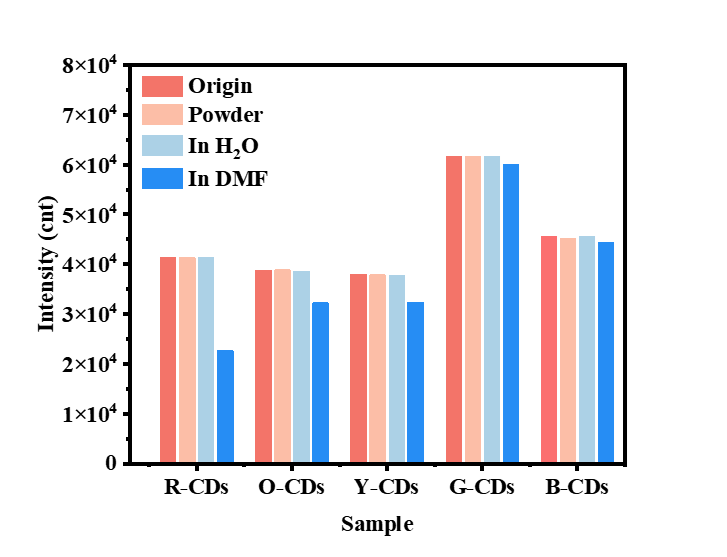 |
| --- |
| Figure S17. Fluorescence intensity variation of multicolor CDs after 10 days under different storage conditions. |

|  |
| --- |
| Figure S18. Curve of total organic carbon of carbon dots vs. ultraviolet irradiation time. |

|  |
| --- |
| Figure S19. XPS spectra of products obtained from R-CDs after 2 h photoreaction in different solvents. |

| 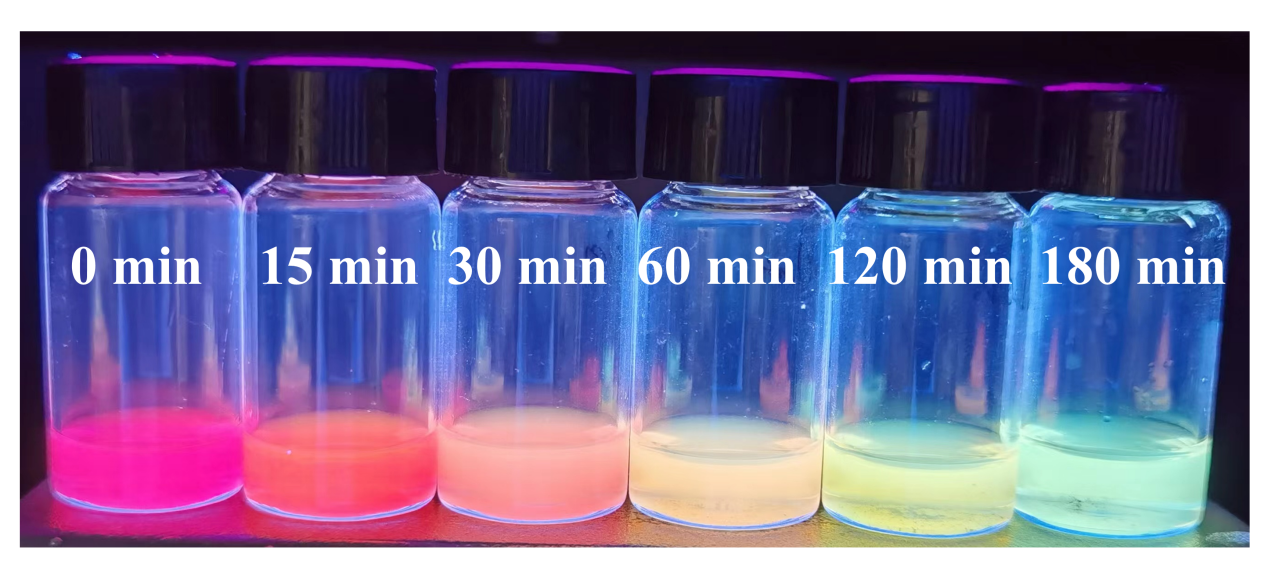 |
| --- |
| Figure S20. Images of R-CDs with different ultrasonication process times under UV light. |

|  |
| --- |
| Figure S21. Fluorescence spectra of R-CDs after different ultrasonic treatment times. |

| **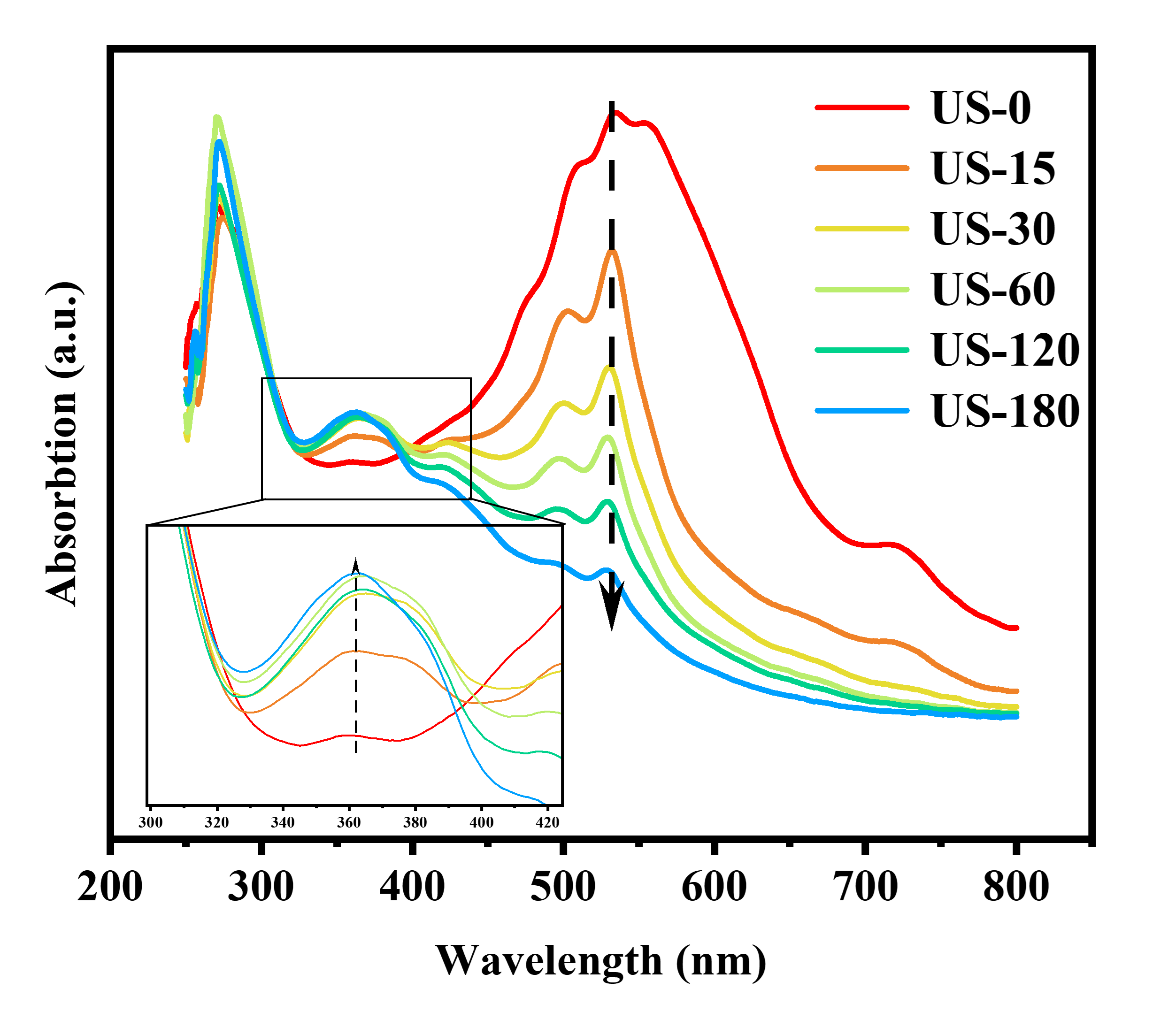** |
| --- |
| Figure 22. Uv-vis absorption spectra of R-CDs after different ultrasonic treatment times. |

| **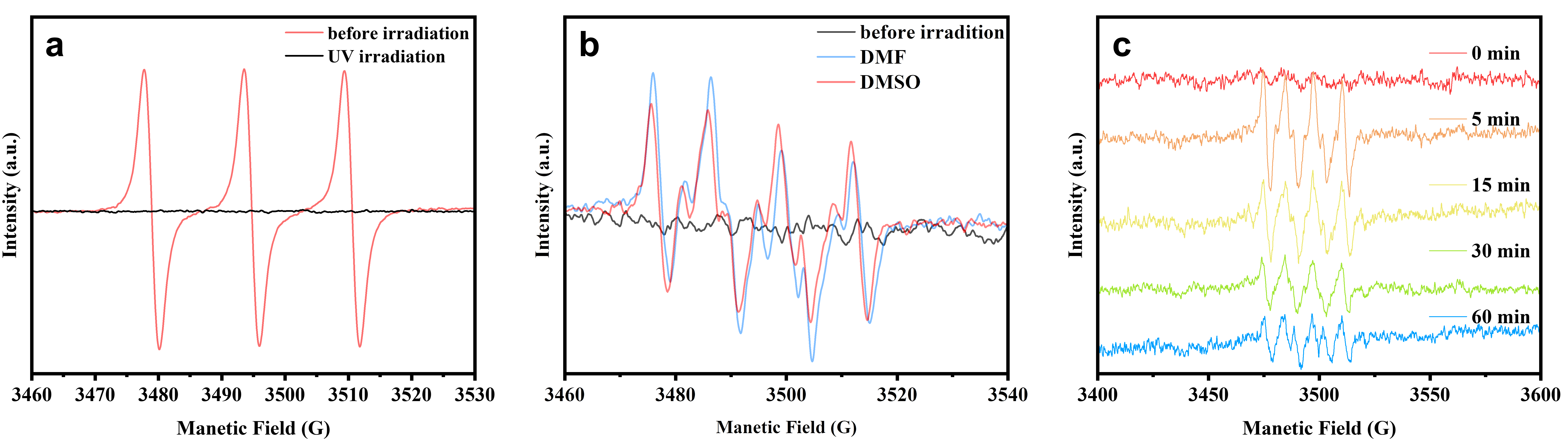** |
| --- |
| Figure 23. a) Epr signal of TEMPO radical after adding R-CDs. b) Signal of DMPO-O_2_^-^ upon addition of r-cds in different solvents. c) DMPO-O_2_^-^ EPR signals of R-CDs in DMF under different irradiation times. |

| **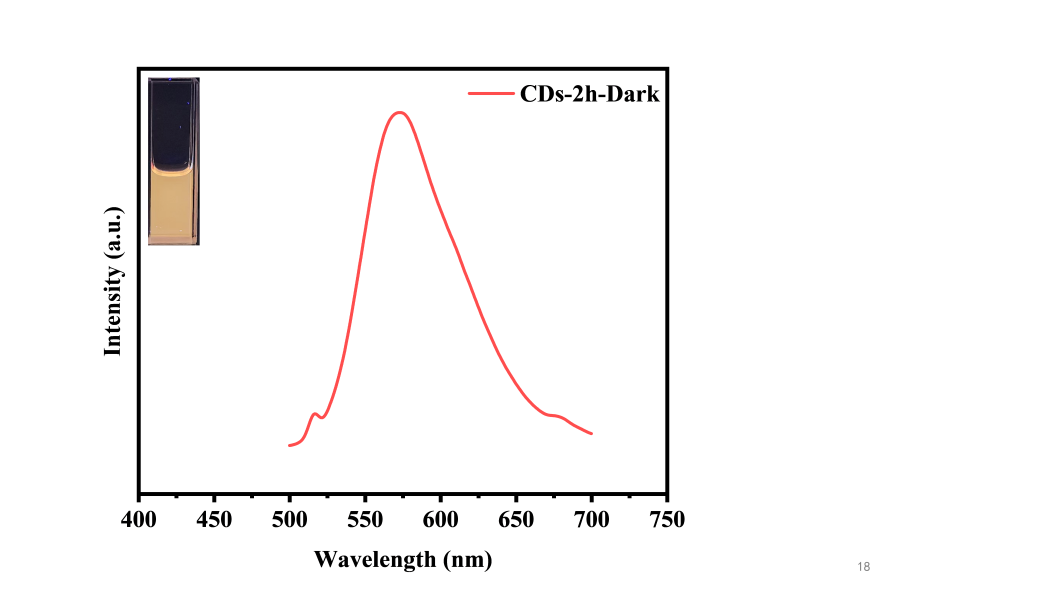** |
| --- |
| Figure S24. Fluorescence spectra and photographs under ultraviolet light of CDs-2h-Dark. |

| **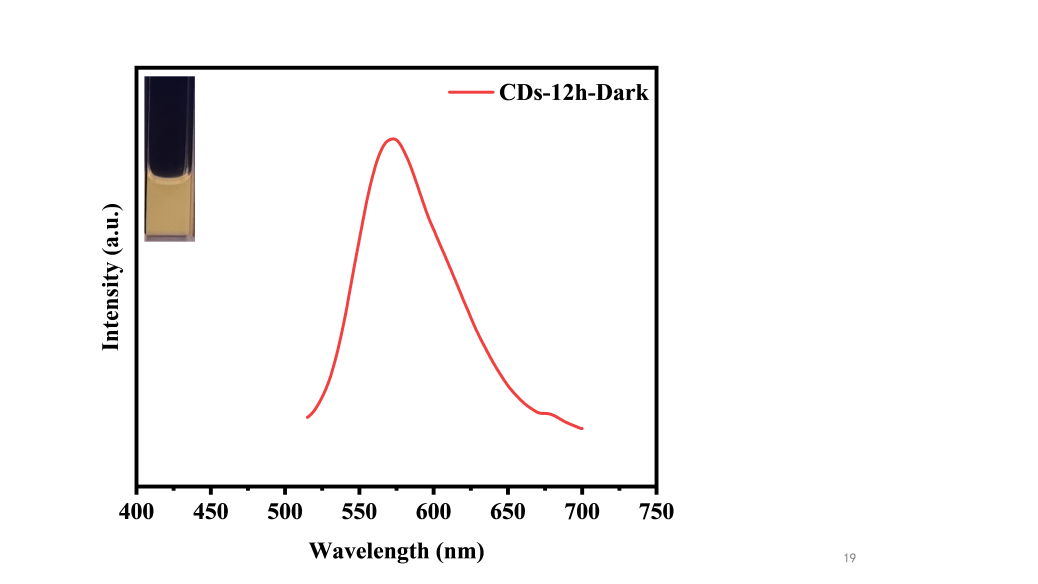** |
| --- |
| Figure S25. Fluorescence spectra and photographs under ultraviolet light of CDs-12h-Dark. |

| 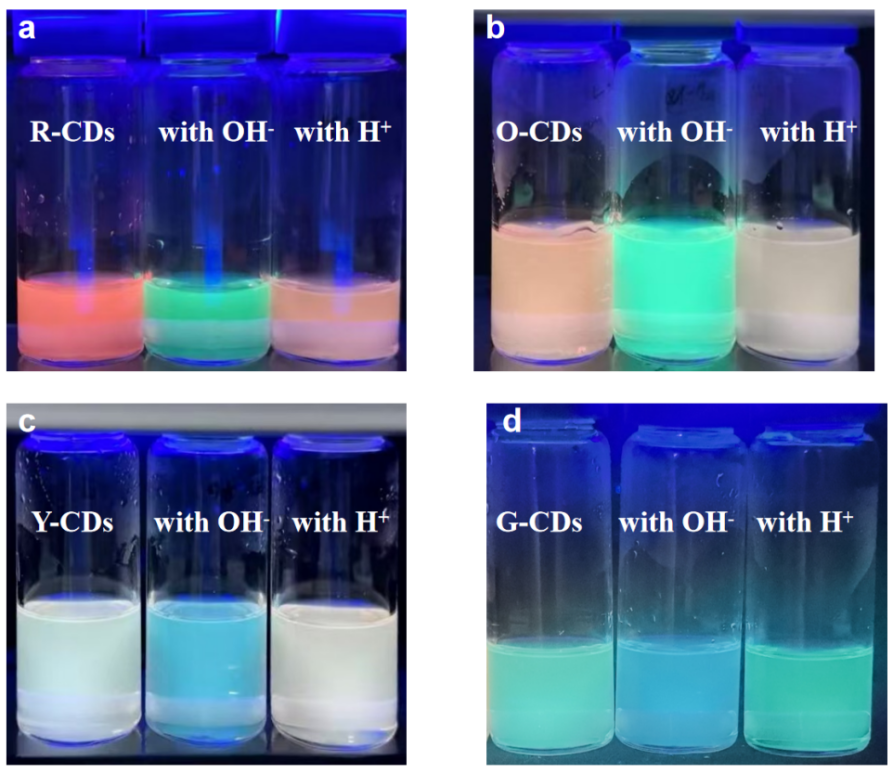 |
| --- |
| Figure S26. Photographs of R-CDs (a), O-CDs (b), Y-CDs (c) and G-CDs (d) in acidic and alkaline environments under UV light. |

| 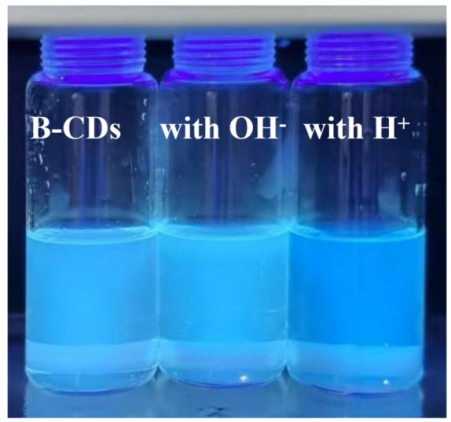 |
| --- |
| Figure S27. Photographs of B-CDs in acidic and alkaline environments under UV light. |

| **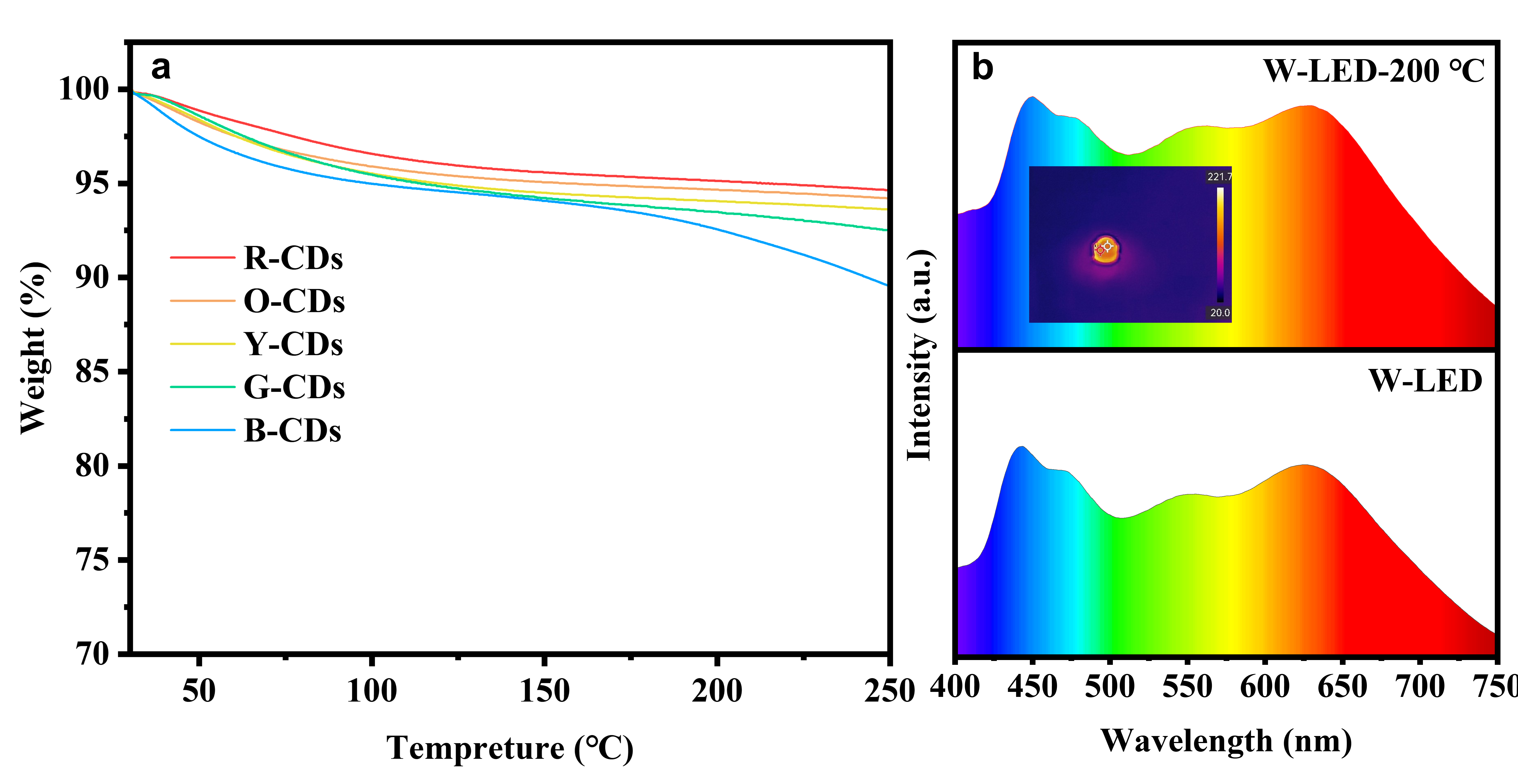** |
| --- |
| Figure S28. a) TGA curves of multicolor CDs. b) Emission spectra of W-LED at elevated temperatures (inset: thermal image). |

| 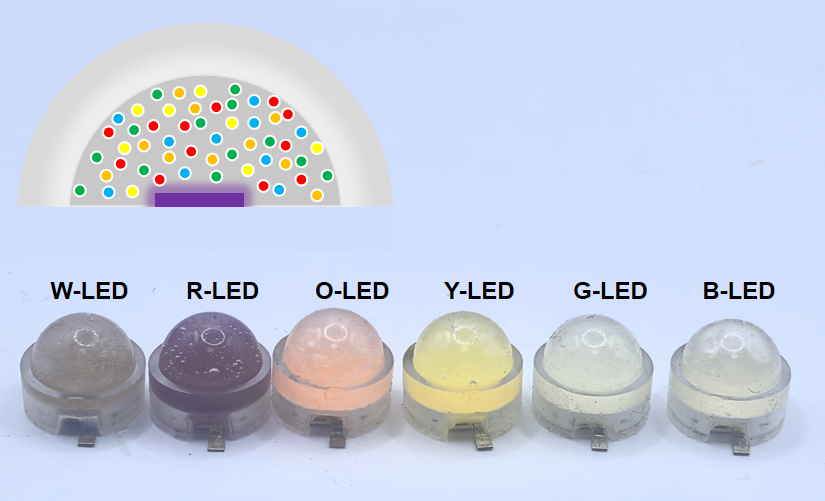 |
| --- |
| Figure S29. Photographs and structural schematic diagrams of the CDs-based LEDs. |

| 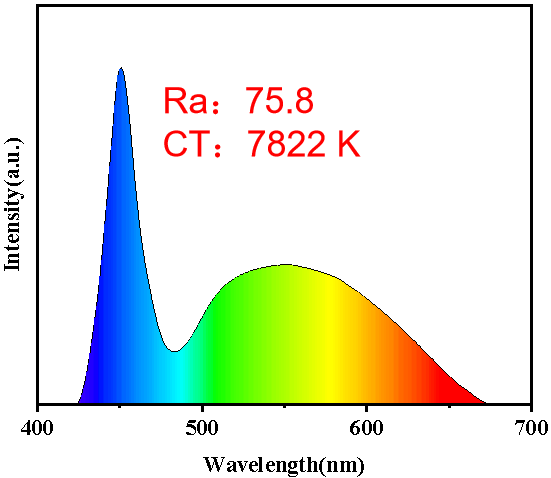 |
| --- |
| Figure S30. Spectrum of Commercial White LED. |

|  |
| --- |
| Figure S31. Variations of CRI and CCT of W-LED as a function of operating time. |

| **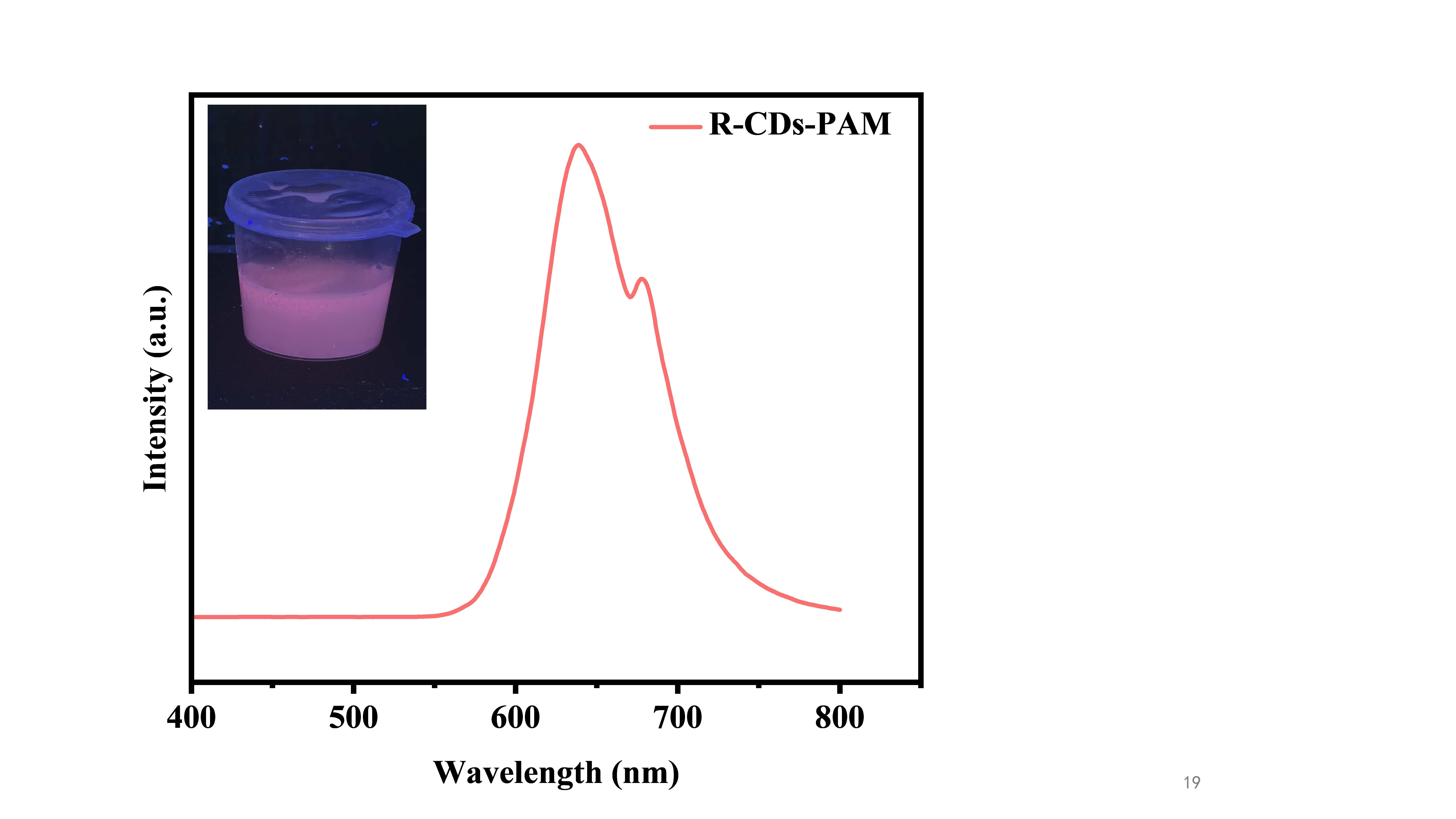** |
| --- |
| Figure S32. Fluorescence spectrum of R-CDs-PAM gel and its photograph under UV lamp illumination. |

| 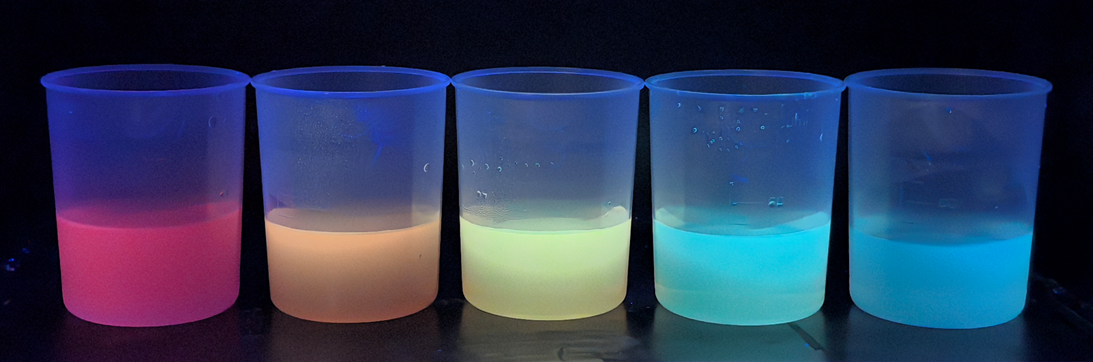 |
| --- |
| Figure S33. Photograph of the muticolor CDs-PVA gel under ultraviolet light. |
